# Wall teichoic acid substitution with glucose governs phage susceptibility of *Staphylococcus epidermidis*

**DOI:** 10.1128/mbio.01990-23

**Published:** 2024-03-12

**Authors:** Christian Beck, Janes Krusche, Anna Notaro, Axel Walter, Lara Kränkel, Anneli Vollert, Regine Stemmler, Johannes Wittmann, Martin Schaller, Christoph Slavetinsky, Christoph Mayer, Cristina De Castro, Andreas Peschel

**Affiliations:** 1 Cluster of Excellence “Controlling Microbes to Fight Infections (CMFI)”, University of Tübingen, Tübingen, Germany; 2 Interfaculty Institute of Microbiology and Infection Medicine Tübingen, Infection Biology, University of Tübingen, Tübingen, Germany; 3 German Center for Infection Research (DZIF), Partner Site Tübingen, Tübingen, Germany; 4 Department of Agricultural Sciences, University of Naples, Naples, Italy; 5 Interfaculty Institute of Microbiology and Infection Medicine Tübingen, Organismic Interactions/Glycobiology, University of Tübingen, Tübingen, Germany; 6 Electron-Microscopy, Department of Dermatology, University Hospital Tübingen, Tübingen, Germany; 7 Leibniz Institute, DSMZ-German Collection of Microorganisms and Cell Cultures, Braunschweig, Germany; 8 Pediatric Surgery and Urology, University Children's Hospital Tübingen, University of Tübingen, Tübingen, Germany; 9 Department of Chemical Sciences, University of Naples, Naples, Italy; Institut Pasteur, Paris, France; National Institute of Allergy and Infectious Diseases Division of Intramural Research, Bethesda, USA

**Keywords:** coagulase-negative staphylococci, *Staphylococcus epidermidis*, wall teichoic acid, bacteriophages, glycosyltransferase

## Abstract

The species- and clone-specific susceptibility of *Staphylococcus* cells for bacteriophages is governed by the structures and glycosylation patterns of wall teichoic acid (WTA) glycopolymers. The glycosylation-dependent phage-WTA interactions in the opportunistic pathogen *Staphylococcus epidermidis* and in other coagulase-negative staphylococci (CoNS) have remained unknown. We report a new *S. epidermidis* WTA glycosyltransferase TagE whose deletion confers resistance to siphoviruses such as ΦE72 but enables binding of otherwise unbound podoviruses. *S. epidermidis* glycerolphosphate WTA was found to be modified with glucose in a *tagE*-dependent manner. TagE is encoded together with the enzymes PgcA and GtaB providing uridine diphosphate-activated glucose. ΦE72 transduced several other CoNS species encoding TagE homologs, suggesting that WTA glycosylation via TagE is a frequent trait among CoNS that permits interspecies horizontal gene transfer. Our study unravels a crucial mechanism of phage-*Staphylococcus* interaction and horizontal gene transfer, and it will help in the design of anti-staphylococcal phage therapies.

**IMPORTANCE:**

Phages are highly specific for certain bacterial hosts, and some can transduce DNA even across species boundaries. How phages recognize cognate host cells remains incompletely understood. Phages infecting members of the genus *Staphylococcus* bind to wall teichoic acid (WTA) glycopolymers with highly variable structures and glycosylation patterns. How WTA is glycosylated in the opportunistic pathogen *Staphylococcus epidermidis* and in other coagulase-negative staphylococci (CoNS) species has remained unknown. We describe that *S. epidermidis* glycosylates its WTA backbone with glucose, and we identify a cluster of three genes responsible for glucose activation and transfer to WTA. Their inactivation strongly alters phage susceptibility patterns, yielding resistance to siphoviruses but susceptibility to podoviruses. Many different CoNS species with related glycosylation genes can exchange DNA via siphovirus ΦE72, suggesting that glucose-modified WTA is crucial for interspecies horizontal gene transfer. Our finding will help to develop antibacterial phage therapies and unravel routes of genetic exchange.

## INTRODUCTION

*Staphylococcus epidermidis* is one of the most abundant colonizers of mammalian skin and nasal epithelia (1, 2). Some nosocomial *S. epidermidis* clones also cause invasive infections, in particular biofilm-associated infections on catheters or artificial implants such as hip and knee joints or heart valves (3, 4). Although *S. epidermidis* is not an as aggressive pathogen as *Staphylococcus aureus*, biofilm-associated infections are difficult to treat and cause a high burden of morbidity and costs for health care systems. Many *S. epidermidis* clones are resistant to beta-lactams and other antibiotics such as linezolid, which further complicates the treatment of *S. epidermidis* infections (1).

The major invasive *S. epidermidis* clones seem to pursue two different virulence strategies. The multilocus sequence type2 (ST2) strains produce particularly strong biofilms (3, 5). In contrast, ST10, ST23, and ST87 clones are only weak biofilm formers, but they express an additional surface molecule that alters their host interaction capacities and leads to a shift from commensal to pathogen behavior (6). Surface properties and host interaction of staphylococci are governed not only by surface proteins but also by cell-wall anchored glycopolymers composed of alditolphosphate-repeating units called wall teichoic acids (WTA) (7, 8). The WTA polymers of *S. epidermidis* and other coagulase-negative staphylococci (CoNS) species have remained a neglected field of research despite their potentially critical role for host colonization and infection. Most *S. epidermidis* clones seem to express WTA composed of glycerolphosphate (GroP)-repeating units (9). A recent study has shown that ST10, ST23, and ST87 strains express an additional *S. aureus*-type WTA composed of ribitolphosphate (RboP)-repeating units, which shapes their interaction with human epithelial and endothelial cells (6).

WTA is also crucial for the binding of virtually all known *Staphylococcus* phages, which use differences in WTA structure to recognize their cognate host species (10). Phages of the sipho- and podovirus groups often not only discriminate between different WTA backbones but also between different types of backbone glycosylation. Most Firmicutes link D-alanine esters and sugar residues to GroP- or RboP-repeating units (7, 8). Variation in glycosylation for instance by N-acetylglucosamine (GlcNAc) in alpha or beta configuration or N-acetylgalactosamine (GalNAc) has been found to govern the susceptibility patterns of *S. aureus* strains for different phages (11–14). The group of broad-host range myoviruses, however, requires WTA for binding but does not discriminate between RboP and GroP WTA and does not require WTA glycosylation (15–17).

WTA-phage interaction is of importance for phage-therapeutic strategies, which have gained increasing attention recently (3, 18). Moreover, they are critical for interspecies horizontal gene transfer via transducing bacteriophages (19). Such transduction events have led to the transfer of resistance and virulence genes into the genomes of *S. aureus* and other species, thereby allowing, for instance, the evolution of methicillin-resistant *S. epidermidis* (MRSE) and methicillin-resistant *S. aureus* (MRSA) (20, 21). Despite the critical role of WTA in these processes, the biosynthesis, composition, and glycosylation of the canonical *S. epidermidis* WTA has not been studied.

Here, we demonstrate that *S. epidermidis* strain 1457 (ST86), which has been isolated from a central venous catheter infection (22), glycosylates its GroP-WTA with glucose, and we identify the WTA glucosyltransferase gene *tagE. S. epidermidis tagE* mutants showed complex changes in phage susceptibility patterns including both the loss and the acquisition of susceptibility to certain phages, some of which we found to be capable of transducing plasmid DNA between different CoNS species.

## RESULTS

### Disruption of a putative glycosyl transferase gene cluster confers resistance to phage ΦE72

Several new phages with the capacity to infect *S. epidermidis* have been reported recently (23, 24). Some of them have the capacity to transduce DNA between different *S. epidermidis* lineages, raising the questions of which bacterial target structures are recognized by the phages’ binding proteins and how universal these target structures may be among different clones of *S. epidermidis* and other CoNS. As most *S. aureus* phages recognize the sugar modifications of WTA (11–13, 25), it was tempting to speculate that glycosylated GroP-WTA is also required for the binding of *S. epidermidis* phages. However, the enzymes responsible for WTA glycosylation in *S. epidermidis* have remained unknown, and it has also remained elusive, which glycosylation patterns can be found on *S. epidermidis* GroP-WTA. To elucidate the WTA glycosylation pathways of *S. epidermidis* and explore its impact on phage interaction, we set out to identify and inactivate the responsible enzyme genes.

**Fig 1:**
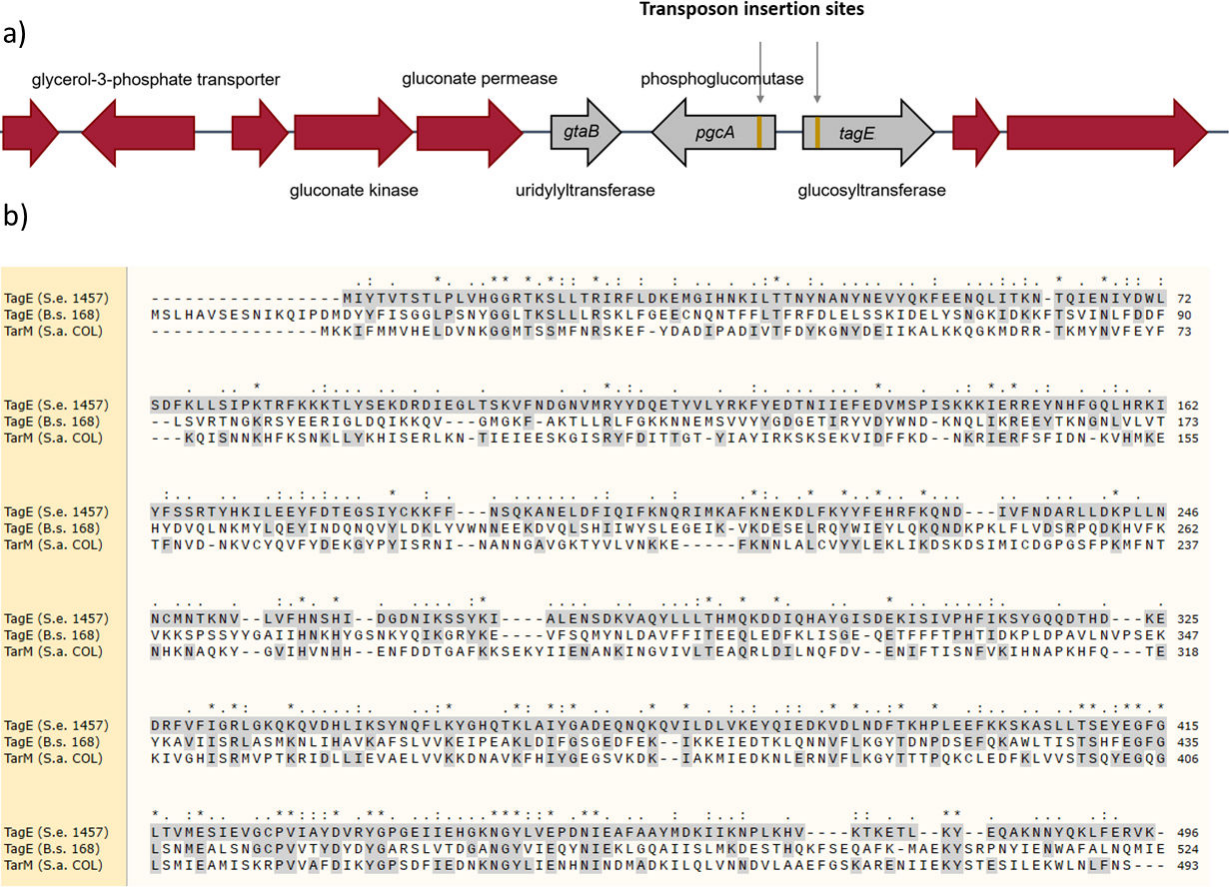
The *tagE* gene encodes a glycosyltransferase in *S. epidermidis*. (**a**) Genetic locus identified by transposon mutagenesis contains the *S. epidermidis tagE*, *pgcA*, and *gtaB* homologs. Transposon insertion sites are labeled in gold. (**b**) MUSCLE alignment of *S. epidermidis* TagE with *B. subtilis* TagE and *S. aureus* TarM protein sequences.

A library of transposon mutants of *S. epidermidis* 1457 was created using a xylose-inducible Himar1 transposase (26) and incubated with phage ΦE72, which is known to infect and multiply in strain 1457 (23). Several isolates, which were resistant to ΦE72, were identified. However, all these isolates had the transposon integrated at only two distinct positions in two adjacent genes of unknown function (Fig. 1a, 2a and d). The two genes were not in the vicinity of other WTA-biosynthesis-related genes, but their gene products shared similarities with glycosylation-related enzymes. The amino acid sequence of the gene B4U56_RS02220 product was 46% similar to TagE of *Bacillus subtilis*, which glycosylates GroP-WTA with glucose residues (27) and 48% similar to TarM of *S. aureus*, which glycosylates RboP-WTA with GlcNAc (Fig. 1b) (28). The adjacent gene B4U56_RS02215 encodes a protein with 59% similarity to the phosphoglucomutase PgcA of *B. subtilis*, which isomerizes glucose-6-phosphate to yield glucose-1-phosphate (29). In addition, the product of gene B4U56_RS02210, next to *pgcA*, was 85% similar to the GtaB enzyme of *B. subtilis* generating uridine diphosphate glucose (UDP-glucose) from glucose-1-phosphate and UTP (30). Both, PgcA and GtaB are required for glycosylation of GroP-WTA with glucose via TagE in *B. subtilis* (31), although the two genes are not encoded together with *tagE* in *B. subtilis* (32, 33). We assumed that the three enzymes might cooperate in *S. epidermidis* to activate and attach glucose to GroP-WTA.

**Fig 2:**
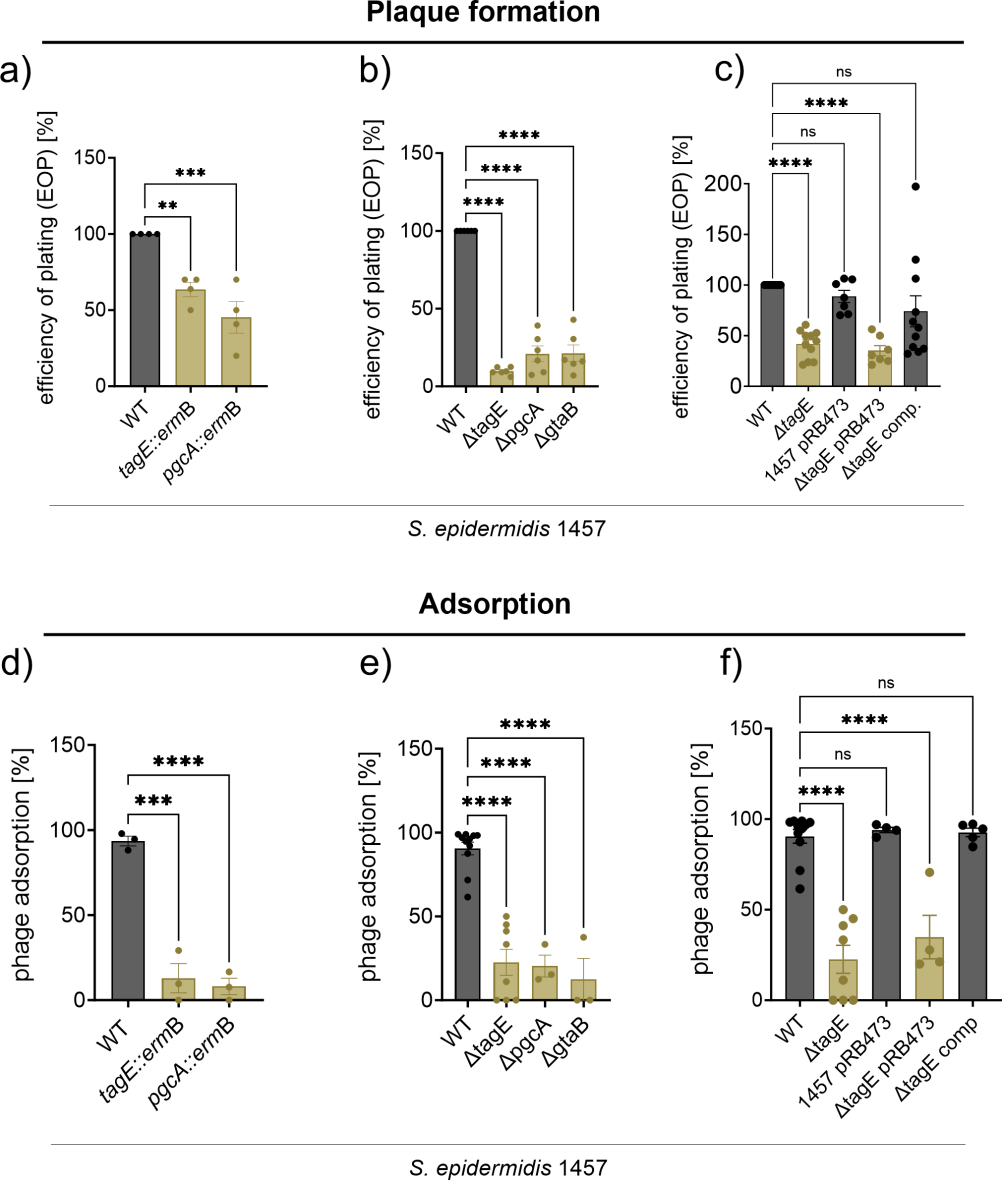
Efficiency of plating (EOP) (**a and b**) and binding (**d and e**) of ΦE72 is reduced on the *tagE*, *pgcA*, and *gtaB* deletion mutants, compared to the *S. epidermidis* wild type (WT). This defect can be restored by complementing the *tagE* mutant with the genetic locus containing *tagE*, *pgcA*, and *gtaB* on plasmid pRB473 (**c and f**). The data represent the mean ± SEM of at least three independent experiments. Ordinary one-way analysis of variance (ANOVA) was used to determine statistical significance vs *S. epidermidis* 1457 WT, followed by Dunnett’s multiple comparisons tests, indicated as: not significant (ns), ^**^P < 0.01, ^***^P < 0.001, and ^****^P < 0.0001.

### *S. epidermidis* TagE is responsible for glucose addition to GroP-WTA

The three *S*. *epidermidis* genes were renamed according to the corresponding *B. subtilis* genes *tagE*, *gtaB*, and *pgcA*. All three genes were inactivated by targeted deletion to confirm their roles in phage susceptibility. The three deletion mutants, like the two transposon mutants, showed reduced susceptibility to ΦE72 infection, and complementation of the *tagE* mutant with a plasmid-encoded copy of the gene locus restored wild-type (WT) level ΦE72 susceptibility (Fig. 2c and f). The various transposon and targeted deletion mutants were approximately threefold less susceptible to ΦE72 infection but were not completely resistant, suggesting that the phage may have additional, albeit less effective, ways to interact with *S. epidermidis* 1457. In a similar way, and even more pronounced, the mutants had retained only limited capacities to bind ΦE72 particles in liquid media (Fig. 2d through f; Fig. S1).

**Fig 3:**
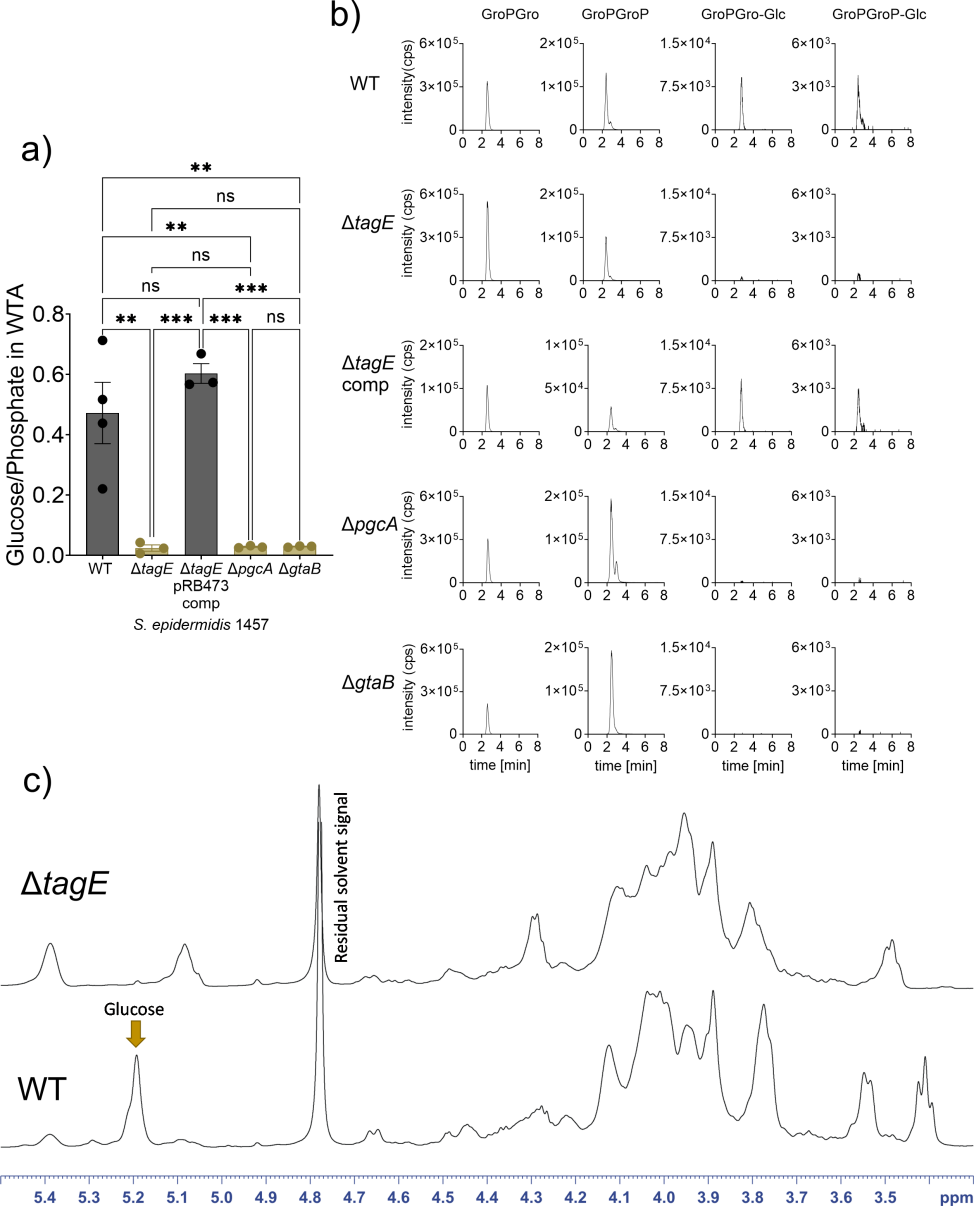
WTA analysis of the *S. epidermidis* mutants ∆*tagE*, ∆*pgcA*, ∆*gtaB*, and of ∆*tagE* containing the pRB473 plasmid carrying *tagE*, *pgcA*, and *gtaB* genes for complementation. (**a**) Ratio of glucose per phosphate content of WTA was measured enzymatically. (**b**) HPLC-MS: Extracted ion chromatograms of GroP-Gro [(M - H)^-^ = 245.0432] and GroP-GroP [(M - H)^-^ = 325.0095] with [GroP-Gro-Glc; (M - H)^-^ = 407.096] [GroP-GroP-Glc; (M - H)^-^ = 487.0623] or without glucose substitution. (**c**) ^1^H NMR spectra reveal D-glucose on WTA of the *S. epidermidis* 1457 WT (at the C2-position of GroP), while the deletion of *tagE* results in the absence of glucose on WTA. For (a), data represent the mean ± SEM of at least three independent experiments. Ordinary one-way ANOVA was used to determine statistical significance, followed by Tukey’s multiple comparisons tests, indicated as: not significant (ns), ^**^P < 0.01, and ^***^P < 0.001.

WTA isolated from 1457 WT contained substantial amounts of glucose when analyzed by an enzymatic glucose assay indicating that ca. 50% of the GroP-WTA-repeating units are modified with glucose (Fig. 3a). In contrast, none of the WTA samples of any of the *tagE*, *gtaB*, or *pgcA* mutants was found to contain glucose. High-performance liquid chromatography coupled to a mass spectrometry detector (HPLC-MS) and nuclear magnetic resonance (NMR) spectroscopy confirmed the presence of glucose-substituted GroP-repeating units in the WT and the absence of glucose in the mutants (Fig. 3b and c; Fig. S2). These findings reflect earlier reports on the presence of glucose on *S. epidermidis* GroP-WTA (9), and they confirm that the PgcA-GtaB-TagE pathway is required for GroP-WTA glycosylation with glucose. NMR analysis indicated that the glucose units are α-configured at the anomeric center and attached to the C2-position of GroP. About 15% of the glucose residues are modified with D-alanine at the O6-position of glucose [Fig. 3c; supplemental material (NMR extended description); Table S3; Fig. S7]. The α-configuration is reminiscent of the configuration of GlcNAc on RboP-WTA introduced by the TagE-related TarM in *S. aureus* (28).

The absence of glucose on GroP-WTA in the ∆*tagE* mutant did not alter biofilm formation by *S. epidermidis* 1457 (Fig. S3). Moreover, no differences in growth kinetics (Fig. S1), cell wall thickness, or cell shape (Fig. S4) were observed in the mutants, indicating that the absence of glucose on GroP-WTA has no major impact on the overall cellular properties of the *S. epidermidis* surface.

Preincubation of ΦE72 with purified cell wall effectively prevented ΦE72 infection of *S. epidermidis* only if glucose was present on the attached WTA (Fig. 6a). In contrast, purified WTA had no such effects (Fig. S5), suggesting that ΦE72 only binds to its GroP WTA receptor when it is modified with glucose and embedded in the cell wall matrix.

**Fig 4:**
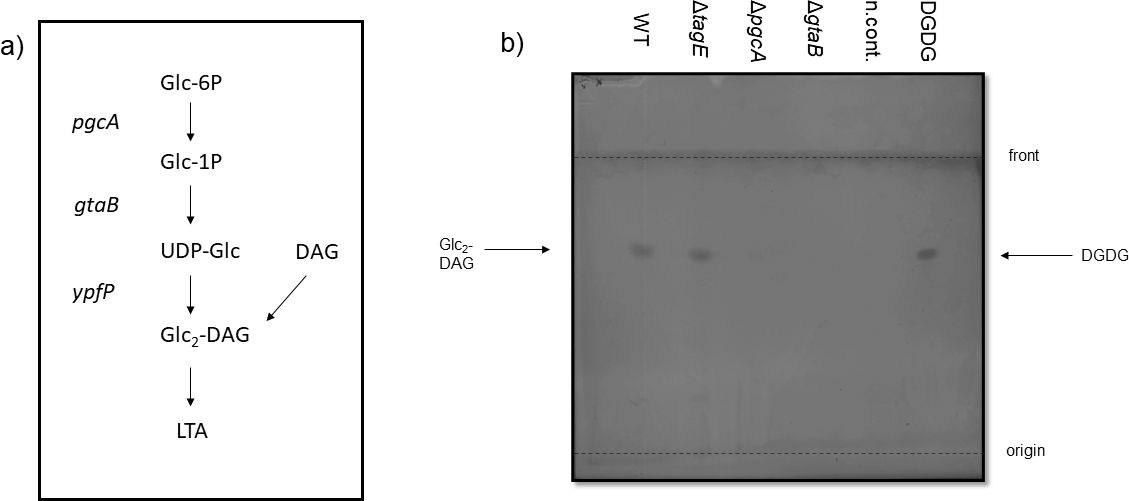
Glycolipid detection by thin layer chromatography (TLC). (**a**) LTA glycolipid biosynthesis pathway as described for *S. aureus* and *B. subtilis* [adapted from reference (37)]. (**b**) Glycolipid detection on a TLC plate stained with α-naphthol/sulfuric acid. For positive control, 5 µg of digalactosyldiacylglycerol (DGDG) was used, while the solvent methanol/chloroform (1:1) was used as negative control (n.cont.). One representative experiment of three independent experiments is shown.

UDP-glucose generated by PgcA and GtaB is also required for the biosynthesis of the glycolipid diglucosyldiacylglycerol (DGlcDAG), which serves as anchor structure for lipoteichoic acid (LTA) polymers in *B. subtilis* and many other Firmicutes (Fig. 4a) (34–36). However, DGlcDAG is not essential for LTA biosynthesis because mutants lacking glycolipids still produce LTA attached to phosphatidylglycerol lipids (36, 37). The *S. epidermidis pgcA* and *gtaB* mutants, but not the *tagE* mutant, also lacked DGlcDAG, which was present in the parental strain (Fig. 4b), indicating that DGlcDAG is synthesized in *S. epidermidis* by the same pathway as in *B. subtilis* and *S. aureus*.

### Lack of WTA glucose impairs the binding of known *S. epidermidis* siphoviruses but promotes the binding of podoviruses

**Fig 5:**
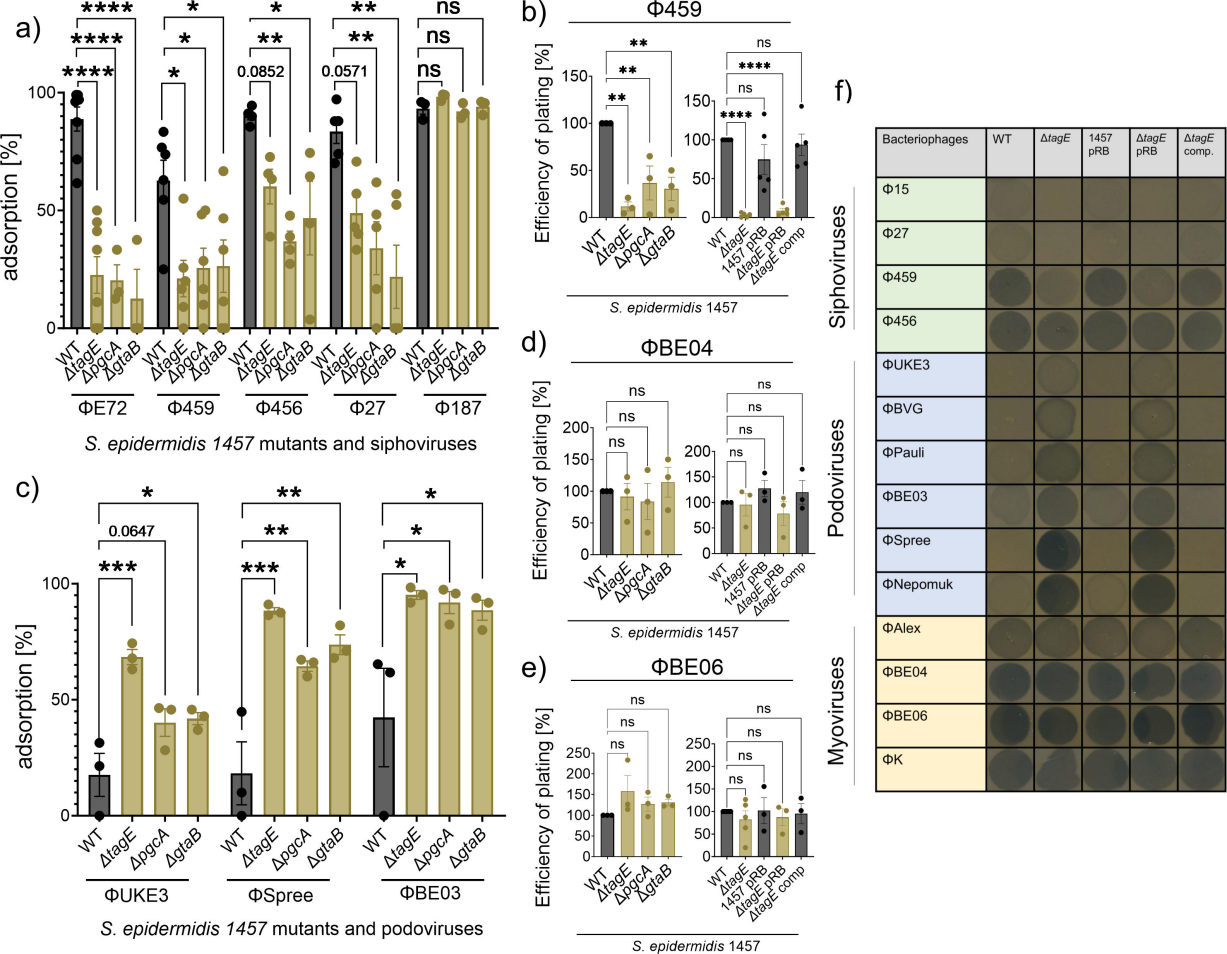
TagE-glycosylated WTA increases the binding of siphoviruses but reduces podovirus binding. WTA glycosylation-deficient mutants of *S. epidermidis* show decreased binding of ΦE72-related siphoviruses Φ459, Φ456, and Φ27 (**a**) but increased binding of the podoviruses ΦUKE3, ΦSpree, and ΦBE03 (**c**), while the GroP-GalNAc-specific siphovirus Φ187 still shows strong binding (**a**). WTA glycosylation-deficient mutants of *S. epidermidis* show less plaque formation by ΦE72-related siphovirus Φ459 (**b**), while plaque formation by the myoviruses ΦBE04 (**d**) and ΦBE06 (**e**) remains unchanged. (**f**) Lysis zones by siphoviruses decrease in the absence of *tagE* but increase for podoviruses. Myoviruses show the formation of lysis zones independent of the presence or absence of *tagE* [pRB = pRB473 (empty vector control); comp = complementation with *tagE*, *gtaB*, and *pgcA* genes]. The data represent the mean ± SEM of at least three independent experiments. Ordinary one-way ANOVA was used to determine statistical significance vs *S. epidermidis* 1457 WT, followed by Dunnett’s multiple comparisons tests, indicated as: not significant (ns), *P < 0.05, **P < 0.01, ***P < 0.001, and ****P < 0.0001. *P*-values are shown if appropriate.

Several other phages in addition to ΦE72 were analyzed for the impact of GroP-WTA glucose modification on phage binding and infection. The ΦE72-related siphoviruses Φ456,Φ459, and Φ27, which are known to bind to *S. epidermidis* 1457 (23), showed reduced binding to the *pgcA*, *gtaB*, and *tagE* mutants compared to the WT, but the reduction was less pronounced as for ΦE72 (Fig. 5a and f). Φ459 was equally reduced in its capacities to propagate in the mutants as ΦE72 (Fig. 5b). Despite their capacity to bind *S. epidermidis* 1457, Φ27 and Φ456 did not form clear plaques on WT or mutant strains. Two recently isolated myoviruses of the genus sepunavirus, ΦBE04 and ΦBE06 (38), showed no reduction in their ability to bind and infect the mutants, suggesting that these myoviruses are not dependent on glucose-modified GroP-WTA (Fig. 5d and e). This resembles the infection behavior of myovirus ΦK, for which the WTA backbone is sufficient for binding to *S. aureus* (13).

**Fig 6:**
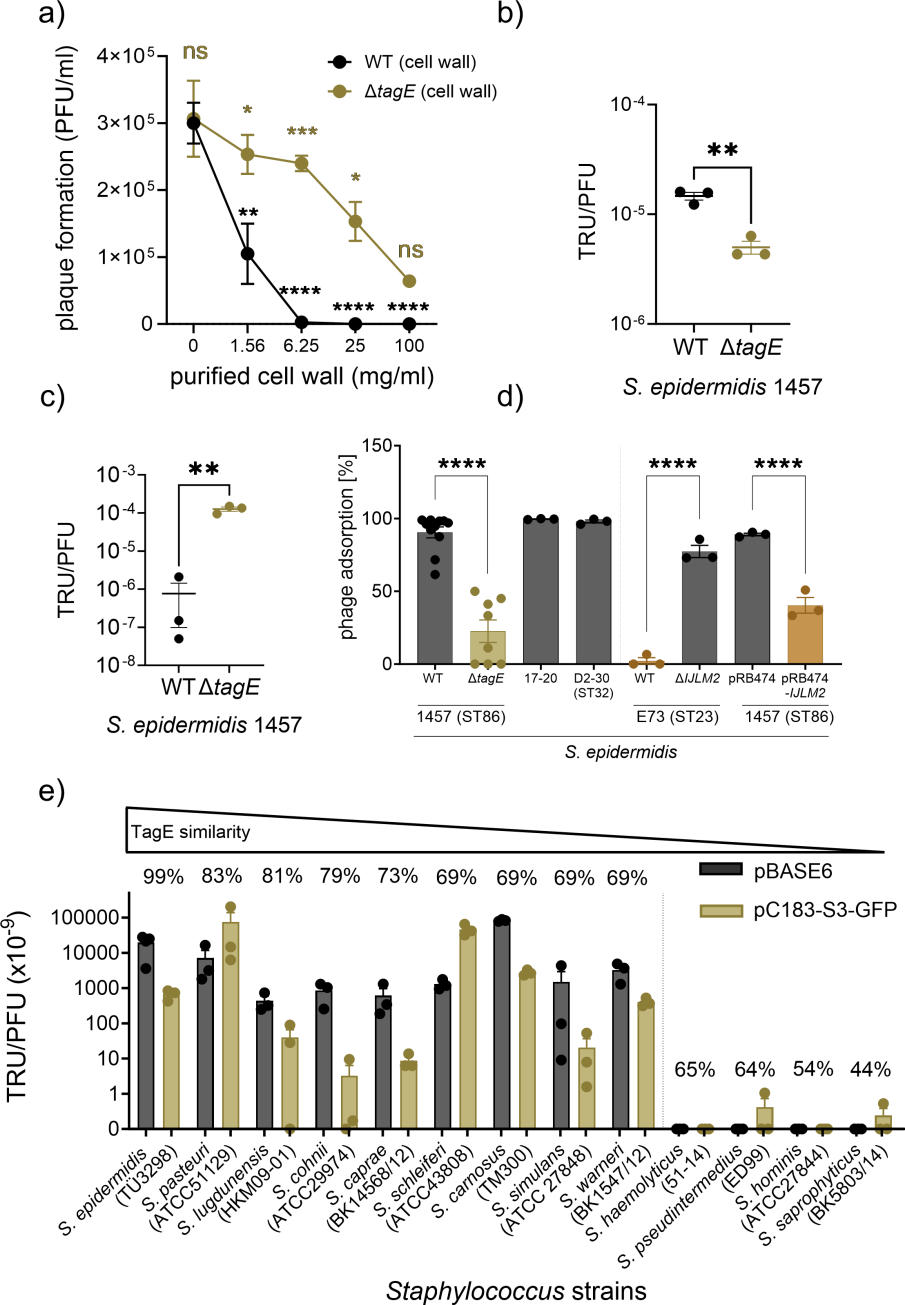
TagE-modified WTA determines horizontal gene transfer between CoNS. (**a**) The purified cell wall of *S. epidermidis* 1457 with glucose-modified GroP-WTA (black) prevents plaque formation by ΦE72 more efficiently than in the absence of glucose (gold). (**b**) Transduction of the plasmid pRB473 by ΦE72 is decreased if the receiving strain lacks WTA modification with glucose. (**c**) Transduction of pRB473 with Φ187 is increased in the absence of glucose on GroP-WTA. (**d**) ΦE72 binds to different strains of *S. epidermidis*, but binding is prevented by RboP expression encoded by the *tarIJLM2* cluster. (**e**) ΦE72-mediated transduction of pBASE6 or pC183-S3-GFP to CoNS depends on high TagE homology. Strain names are given in brackets. The data represent the mean ± SEM of at least three independent experiments. For (a), two-way ANOVA was used to determine statistical significance comparing unsupplemented control vs WT cell wall supplementation (black) and supplementation of WT cell wall vs Δ*tagE* cell wall (gold), followed by Tukey’s multiple comparisons tests. For (b) and (c), unpaired *t* tests were used to determine statistical significance vs *S. epidermidis* 1457 WT, and for (d), ordinary one-way ANOVA was used to determine statistical significance vs *S. epidermidis* 1457 WT, followed by Tukey’s multiple comparisons tests. Statistical significance is indicated as: not significant (ns), *P < 0.05, **P < 0.01, ***P < 0.001, and ****P < 0.0001.

Several other phages, which bind *S. epidermidis* 1457 but cannot replicate in this strain, behaved differently. Siphovirus Φ187, which is only distantly related to ΦE72 and requires GroP-WTA modified with GalNAc for infection of target cells (25), still bound to the GroP-WTA glucose-deficient mutants (Fig. 5a), indicating that the GroP-WTA glucose modifications are not necessary for Φ187 binding. Φ187 even showed higher plasmid transduction efficiency in the absence of GroP-WTA glucose residues (Fig. 6c). Furthermore, the podoviruses ΦUKE3, ΦSpree, and ΦBE03 (38) exhibited strongly increased binding to the *pgcA*, *gtaB*, and *tagE* mutants compared to the WT (Fig. 5c and f), indicating that these phages are attenuated for binding in the presence of glucose residues on GroP-WTA. Thus, the GroP-WTA glucose residues are important for most of the known *S. epidermidis* phages, albeit in quite different ways, depending on the individual phage.

### The presence of *tagE* in the genomes of CoNS species corresponds to the capacity of ΦE72 to transduce these species

The *tagE* gene was found in all available *S. epidermidis* genomes (218), 212 of which encoded a full-length DNA sequence with more than 96% nucleotide identity, suggesting that the substitution of GroP-WTA with glucose is a general trait in *S. epidermidis*. Accordingly, ΦE72 bound well to the tested *S. epidermidis* strains from at least two different sequence types (ST86 and ST32) with one exception (Fig. 6d). Notably, ΦE72 did not bind to *S. epidermidis* E73 (ST23), which produces RboP-WTA in addition to GroP-WTA (6). However, an E73 *tarIJLM2* mutant lacking RboP-WTA was effectively bound by ΦE72, indicating that the additional RboP-WTA shields the surface of *S. epidermidis* in a way that precludes binding of the phage. This effect was confirmed by the expression of the RboP-WTA encoding gene cluster of strain E73 in the GroP-WTA expressing strain 1457, which resulted in reduced phage binding (Fig. 6d).

GroP-WTA has been reported in several other CoNS species. The nature of the sugar modifications in these species has remained largely unknown, but several CoNS have been reported to contain either glucose, GlcNAc, or GalNAc attached to WTA (9). We succeeded in transducing many different CoNS species via ΦE72 with either the staphylococcal shuttle vector pBASE or the green-fluorescent protein-expressing plasmid pC183-S3 GFP. Some of the available CoNS genomes were found to encode TagE homologs, albeit with different degrees of sequence conservation, ranging from 44% to 83% similarity (Table 1). Those species with TagE similarities above 69% could be transduced by ΦE72, while those with less conserved TagE homologs did not take up DNA from ΦE72 (Fig. 6e), suggesting that only CoNS with highly conserved versions of TagE may glycosylate their GroP-WTA in a similar way as in *S. epidermidis*, while the others may glycosylate either other WTA backbone types or may transfer other sugars. Among the tested species, *Staphylococcus pasteuri*, *Staphylococcus lugdunensis*, *Staphylococcus cohnii*, *Staphylococcus caprae*, *Staphylococcus schleiferi*, *Staphylococcus carnosus*, *Staphylococcus simulans*, and *Staphylococcus warneri* strains were transducible with ΦE72. Isolates of two of these species, *S. cohnii* and *S. warneri*, have indeed previously been described to produce GroP-WTA, which is modified with glucose (9). In contrast to the varying degrees of conservation of *tagE*, the *pgcA* and *gtaB* genes are present in virtually all *Staphylococcus* genomes with high sequence similarity, including *S. aureus*, probably because UDP-glucose is required in all these species for DGlcDAG glycolipid synthesis (37). Among the strains that encode highly conserved TagE homologs, *tagE* was encoded in the vicinity of both *pgcA* and *gtaB* only in *S. pasteuri* and *S. lugdunensis*, in addition to *S. epidermidis* (Table 1). Thus, phage ΦE72 represents a helpful tool for studying WTA properties and an attractive vehicle for interspecies transduction of DNA among members of the genus *Staphylococcus*.

**TABLE 1:** Conservation of TagE homologs in CoNS strains used for transduction, ordered according to amino acid sequence similarity

Species	Strain name	Query cover (%)	Sequence identity (%)	Sequence similarity (%)	*tagE*, *pgcA*, and *gtaB* encoded together
*S. epidermidis*	TÜ3298	100	99	99	Yes
*S. pasteuri*	ATCC51129	100	69	83	Yes
*S. lugdunensis*	HKU09-01	99	64	81	Yes
*S. cohnii*	ATCC29974	99	61	79	No
*S. caprae*	BK14568/12	99	52	73	No
*S. schleiferi*	ATCC43808	98	50	69	No
*S. carnosus*	TM300	99	48	69	No
*S. simulans*	ATCC27848	99	46	69	No
*S. warneri*	BK15472/12	99	48	69	No
*Staphylococcus haemolyticus*	51–14	99	45	65	No
*Staphylococcus pseudintermedius*	ED99	100	43	64	No
*Staphylococcus hominis*	ATCC27844	65	28	54	No
*Staphylococcus saprophyticus*	BK5803/14	85	25	44	No

## DISCUSSION

WTA structures are known to be highly diverse among Firmicutes, often with species- or even clone-specific composition (7, 39). Most *S. epidermidis* produce a WTA type that is entirely different from that of *S. aureus* with a GroP rather than an RboP backbone. This study shows that *S. epidermidis* uses a GroP backbone with unmodified or with alanylated glucose. It remains unclear why *S. epidermidis* and *S. aureus* have developed such entirely different WTA types. The different structures may limit the number of bacteriophages that can infect and harm either one or both species. However, ΦK, one the most lytic bacteriophages, can lyse *Staphylococcus* cells irrespective of the WTA backbone structure, and a recent study has demonstrated that several *Staphylococcus* myoviruses can infect both, *S. aureus* and *S. epidermidis* (40). The differences in WTA may limit infections and concomitant lysogenization or transduction events by specific members of the siphovirus group, which depend much more on a specific WTA backbone and glycosylation type than myoviruses. Notably, the presence of glucose on GroP-WTA prevented adsorption to *S. epidermidis* by all tested podoviruses (ΦUKE3, ΦSpree, and ΦBE03). The number of available *S. epidermidis*-targeting phages is still very limited, which impedes more extensive studies on the susceptibility of *S. epidermidis* WT and WTA mutant strains for different phage types. Discovery programs for the identification of new phages that can infect *S. epidermidis* will help to clarify these questions in the future.

WTA is an important bacterial ligand for host receptors on mammalian immune cells with critical roles in innate immunity (8, 41). WTA glycosylated with GlcNAc can activate the scavenger receptor langerin on skin Langerhans cells (42). *S. aureus* is found on the skin of atopic dermatitis patients eliciting skin inflammation in a process that probably involves WTA-langerin interaction (8). In contrast, *S. epidermidis* cannot activate langerin (42), probably because its GroP-WTA is glycosylated with glucose. It may be advantageous for *S. epidermidis*, one of the most abundant skin-colonizers (1), and for other CoNS to avoid skin inflammation by producing a non-inflammatory WTA type decorated with glucose.

*S. epidermidis* uses the same pathway for GroP-WTA glycosylation with glucose residues as described for *B. subtilis* (29). Activation of glucose via the PgcA and GtaB enzymes yields UDP-glucose as a donor of glucose residues, which are subsequently transferred to the WTA backbone by TagE. Other WTA glycosyltransferases apart from TarM (28), including those transferring glucose to RboP-WTA in *B. subtilis* W23 (TarQ) (7, 11), GlcNAc to RboP-WTA in certain *S. aureus* clones (TarS and TarP) (11, 43), or GalNAc to GroP-WTA in *S. aureus* ST395 (TagN) (25), share no or very low similarity with TagE. However, protein structure prediction with Alphafold 2 revealed that TagE most likely forms a symmetric, propeller-like homotrimer with each monomer divided into the characteristic glycosyltransferase domain and the β-sheets containing trimerization domain as previously described for the well-studied *S. aureus* glycosyltransferase TarM (Fig. S6) (44–46).

In addition to glucose, WTA is usually also modified with D-alanine (39). Since GroP-repeating units have only one free hydroxyl group for substitution with either D-alanine or glucose, it is not surprising that only ca. 50% of the repeating units carried glucose residues. The teichoic acid D-alanylation machinery attaches D-alanine to a variety of different molecules including LTA, RboP-WTA, and GroP-WTA (47). Its limited specificity for acceptor substrates may explain why a minor portion of the glucose residues on *S. epidermidis* GroP-WTA is also alanylated. GroP-repeating units are shorter than RboP-repeating units, which may explain why the additional RboP-WTA polymers of *S. epidermidis* E73 are probably longer and preclude binding of ΦE72 to strain E73. The additional WTA may, therefore, represent a further strategy to interfere with phage infection and with the interaction of other WTA-binding molecules.

Several other CoNS species might produce a similar WTA type as *S. epidermidis* because they encode potential TagE proteins and interact with ΦE72 as shown by successful ΦE72-dependent transduction (Fig. 6e). This is supported by data from Endl et al. (9), showing that *S. warneri* and *S. cohnii*, both of which are transducible with ΦE72, express GroP-WTA with glucose modification. Likewise, *S. haemolyticus* and *S. hominis*, which could not be transduced, have been found not to produce glucose-modified GroP-WTA in the previous study (9). Interspecies horizontal gene transfer via WTA-binding transducing phages appears to be rather common among CoNS and may have contributed to the import of the methicillin-resistance conferring *mecA* gene into *S. epidermidis* and, eventually, to *S. aureus* to create MRSE and MRSA clones (20). It remains mysterious how the barrier for horizontal gene transfer between *S. epidermidis* and *S. aureus* that results from the substantial differences in WTA structure could be overcome. Specific *S. epidermidis* clonal lineages with both, GroP-WTA and *S. aureus*-type RboP-WTA such as ST10, ST23, and ST87 (6) or the *S. aureus* lineage ST395 producing CoNS-type GroP-WTA (14), may represent critical hubs for the exchange of genetic material between the species *S. epidermidis* and *S. aureus*. Several CoNS species encode potential WTA glycosyltransferase homologs with only low or no similarity to TagE. They may produce other WTA backbones or glycosylate their WTA with other sugars.

*S. epidermidis* often causes difficult-to-treat biofilm-based infections on implanted materials, which frequently require surgical replacement (4). Treatment with lytic bacteriophages that could destroy *S. epidermidis* biofilms holds promise for the development of new therapeutic strategies (3, 18). Understanding how phages detect suitable host bacteria and which *S. epidermidis* clones express corresponding phage-binding WTA motives will be important for the success of such strategies. The TagE-mediated WTA glycosylation with glucose might contribute to the narrow host range of lytic podoviruses such as ΦBE03 (38). Accordingly, finding podoviruses, which bind to GroP-WTA glucose might help to develop efficient therapeutic phage cocktails. Moreover, glycosylated WTA is a major antigen for protective antibodies against *S. aureus* (43, 48, 49) and, probably, also *S. epidermidis*. Therefore, it represents a particularly attractive antigen for vaccine development (49). As for phage therapy, the success of such vaccination strategies will depend on in-depth knowledge on the structure and prevalence of WTA glycoepitopes among different *S. epidermidis* lineages. Our study may motivate more extensive investigations on WTA glycoepitopes in different staphylococcal pathogens and commensals.

## MATERIALS AND METHODS

### Bacterial strains and growth conditions

*S. epidermidis* and *S. aureus* strains were cultivated in a basic medium (BM) and incubated at 37°C on an orbital shaker. *Escherichia coli* strains were cultivated in lysogeny broth (LB). Media were supplemented with appropriate antibiotics chloramphenicol (10 µg/mL) or ampicillin (100 µg/mL). *E. coli* DC10b and *S. aureus* PS187 ∆*sauUSI*∆*hsdR* were used as cloning hosts, and *S. epidermidis* 1457 was used for gene deletion studies. Bacteriophages and propagation strains used in this study are listed in Table S1.

### Transposon mutagenesis of *S. epidermidis* strain 1457

The transposon plasmid pBTn described previously (26) was used to create a transposon library in *S. epidermidis* 1457. The features of this temperature-sensitive *E. coli/S. aureus* shuttle vector include a mini-transposon with an erythromycin resistance cassette flanked by inverted repeats from the horn fly transposon and a xylose-inducible transposase Himar1, which can mobilize the mini-transposon and integrate it into the chromosome with no bias for any specific sequence. Transposon library construction has been described in detail before (28). In short, *S. epidermidis* 1457 was transformed with pBTn followed by mobilization of the mini-transposon into the genome upon xylose induction of the transposase. The pBTn plasmid was cured via shifts to nonpermissive temperature.

### Isolation of phage-resistant transposon mutants

To isolate phage-resistant mutants, the transposon mutant library was infected with ΦE72 at a multiplicity of infection (MOI) of at least 100. After incubation for up to 4 h, the cells were centrifuged at 5,000 × g for 10 min and plated on tryptic soy agar (TSA) containing erythromycin. Single colonies of surviving mutants were transferred to fresh TSA agar plates repeatedly. Phage resistance was confirmed by spot assays with ΦE72, and the phage-resistant mutants were treated with 1 µg/mL mitomycin to test for and to exclude lysogeny. To identify the site of transposon insertion, total DNA was isolated, purified with the NucleoSpin tissue kit (Macherey-Nagel, Düren), digested, religated, multiplied with primers erm-For and erm-Rev (Table S2), which anneal to the erythromycin resistance cassette of the mini-transposon, and sequenced.

### Molecular genetic methods

For the construction of the *tagE*, *pgcA*, and *gtaB* mutants in *S. epidermidis* 1457, the pBASE6 *E. coli/S.aureus* shuttle vector was used according to standard procedures (50). For mutant complementation, plasmid pRB473 was used (51). The primers for knockout and complementation plasmid construction are listed in (Table S2). Both pBASE6 and pRB473 containing either the respective up- and downstream fragments for knockout construction (pBASE6) or the complementation sequence (pRB473) were used to transform *E. coli* DC10b and subsequently PS187 ∆*sauUSI*∆*hsdR* by electroporation. The plasmids were subsequently transferred to *S. epidermidis* strain 1457 by transduction with Φ187 using *S. aureus* PS187 ∆*sauUSI*∆*hsdR* as donor strain as described previously (19).

### Phage binding, infection, and transduction assays

Phage spot assays were performed as described previously (14). All applied bacteriophages (Table S1) were propagated in suitable bacterial host strains, and phage lysates were filtered to yield sterile phage suspensions. Test bacteria were cultivated overnight in fresh BM. OD_600_ = 0.1 was adjusted in 5 mL LB soft agar for the preparation of bacterial overlay lawns. Five or 10 µL of phage suspensions were spotted onto the bacterial lawns. After overnight incubation at 37°C for podoviruses and siphoviruses, and 30°C for myoviruses, phage-clearing zones and individual plaques were observed and recorded. Efficiency of plating (EOP) was calculated by dividing the number of plaques formed on the deletion mutants and complemented strains by the number of plaques formed on the *S. epidermidis* 1457 WT. The WT was set to 100% EOP. Plaque formation was inhibited by incubating phage suspensions with different amounts of purified cell wall or WTA for 15 minutes, prior to spotting on the indicator strain.

Phage adsorption efficiency was determined as described previously with minor modifications (14). Briefly, adsorption rates were analyzed by mixing approximately 10^6^ plaque forming units per mL (PFU/mL) in BM supplemented with 4 mM CaCl_2_ with the tested bacteria at an OD_600_ of 0.5 and incubating for 15 min at 37°C. The samples were subsequently centrifuged, and the supernatants were spotted on indicator strains to determine the number of unbound phages in the supernatant. The adsorption rate was calculated by dividing the number of bound phages by the number of input phages.

Transduction experiments were performed as described previously (14). Briefly, 1 mL of exponentially growing cultures of a recipient strain was adjusted to an OD_600_ of 0.5. The cells were sedimented by centrifugation and resuspended in 200 µL of phage buffer containing 0.1% gelatin, 1 mM MgSO_4_, 4 mM CaCl_2,_ 50 mM Tris, and 0.1 M NaCl. A mixture was prepared by combining 200 µL of bacteria in phage buffer with 100 µL of lysates obtained from *S. aureus* PS187 and *S. epidermidis* 1457 donor strains carrying plasmids of choice. Samples were then incubated for 15 min at 37°C, diluted, and plated on chloramphenicol-containing BM agar to count colonies. When *S. epidermidis* 1457 was transduced, sodium citrate was added to a final concentration of 15 mM after the incubation step, and selection plates were supplemented with 20 mM sodium citrate.

### Electron microscopy

*S. epidermidis* 1457 WT, *∆tagE*, *∆pgcA*, and *∆gtaB* were grown until stationary phase and fixed at an OD_600_ of 10 in 200 µL Karnovsky’s fixative (3% formaldehyde and 2.5% glutaraldehyde in 0.1 M phosphate buffer pH 7.4) for 24 h. Samples were then centrifuged at 1,400 × g for 5 min, supernatant was discarded, and pellets were resuspended in approximately 20 µL agarose (3.9%) at 37°C, cooled to room temperature, and cut into small pieces. Postfixation was based on 1.0% osmium tetroxide containing 2.5% potassium ferrocyanide (Morphisto) for 2 h. After following the standard methods, samples were embedded in glycide ether and cut using an ultramicrotome (Ultracut E, Reichert). Ultra-thin sections (30 nm) were mounted on copper grids and analyzed using a Zeiss LIBRA 120 transmission electron microscope (Carl Zeiss) operating at 120 kV.

### Cell wall and WTA isolation

WTA was isolated as described previously (14, 52, 53) with minor modifications. Briefly, bacterial cells from 2 L of overnight cultures were washed and disrupted with glass beads in a cell disrupter (Euler). Cell lysates were incubated at 37°C overnight in the presence of DNase and RNase. SDS was added to a final concentration of 2% followed by ultrasonication for 15  min. Cell walls were washed several times to remove SDS. Purified cell wall was used for further experiments. To release WTA from cell walls, samples were treated with 5% trichloroacetic acid for 4 h at 65°C. Peptidoglycan debris was separated via centrifugation (10 min, 14,500 × g). Determination of phosphate amounts as described previously (53, 54) was used for WTA quantification. Crude WTA extracts were further purified as already described (28). Briefly, the pH of the crude extract was adjusted to five with NaOH and dialyzed against the water with a Slide-A-Lyzer Dialysis Cassette (molecular weight cut-off (MWCO) of 3.5  kDa; Thermo Fisher Scientific). The dialyzed WTA was further lyophilized. For HPLC-MS analysis, 50 µL of 100 mM WTA sample was hydrolyzed with 100 mM NaOH at 60°C for 2 h. For NMR, 10–15 mg lyophilized WTA sample was used. WTA was stored at −20°C. Detailed explanations of the HPLC-MS and NMR methods can be found in the supplemental material’s extended descriptions of detailed methods.

### Enzymatic determination of glucose in the WTA samples

The High Sensitivity Glucose Assay Kit (mak181, Sigma-Aldrich) was used to determine the glucose content in the WTA sample. In a vacuum concentrator at 60°C, 50 µL of dialyzed WTA samples and 50 µL of 1 mM glucose standard solution were dried. The samples and the standard solution each had 100 µL of 0.5 M HCl added to them and then cooked for 2 h in a water bath. The glucose standard was diluted 1:50 resulting in a 10 µM concentration, and different volumes were used to cover a range of 0–100 pmol. Samples were also diluted at least 1:50, and different dilutions of the samples were tested in a 96-well plate. The assay was performed according to the manufacturer’s instructions. The fluorescence intensity was measured at excitation wavelength 535 nm and emission wavelength 587 nm.

### Phage saturation with purified cell wall or WTA

Purified cell wall and WTA concentrations were measured by weighing the cell wall or determination of the phosphate content. 100 µL of approximately 10^5^ PFU/mL ΦE72 for WTA supplementation experiments and 100 µL of approximately 10^6^ PFU/mL ΦE72 for cell wall supplementation experiments were mixed with different concentrations of WTA or cell wall and incubated at 37°C for 15 min while shaking at 300 rpm. Inhibition of plaque formation was measured by spotting the solution on soft agar containing *S. epidermidis* 1457 as indicator strain.

### Glycolipid isolation, TLC, and detection with α-naphthol

The detection of glycolipids was performed similar to a previously described method (37). *S. epidermidis* 1457 and the respective mutants were grown to OD_600_ of 3.5. 5 mL of bacterial suspension were washed and resuspended in 500 µL of 100 mM sodiumacetate (pH 4.7) and transferred into glass vials. 500 µL chloroform and 500 mL methanol were added, and the mixture was vortexed vigorously. The samples were centrifuged at 4,600 × g for 20 min at 4°C, and the lower phase was dried overnight and resuspended in 25 µL methanol and chloroform in a 1:1 ratio. The whole sample was applied to a high-performance thin-layer chromatography (HPTLC) silica gel 60 plate (10 × 10 cm; Merck) with a Hamilton syringe. A positive control containing 5 µg digalactosyldiacylglycerol (DGDG, Sigma-Aldrich) was used. A Linomat 5 (Camag) and an auto-developing chamber (Camag) were used to apply the sample to the TLC plate and to run it with a solvent containing 65:25:4 (vol/vol/vol) chloroform/methanol/H_2_O. The dried TLC plate was sprayed with 3.2% α-naphthol in methanol/H_2_SO_4_/H_2_O 25:3:2 (vol/vol/vol), and the glycolipids were visualized by heating the plate at 110°C for a few minutes.

### *In silico* analysis

All statistical analyses were performed with Graph Pad Prism 9.2.0 (GraphPad Software, La Jolla, USA). Multiple sequence alignment was performed with SnapGene 5.3.2 using MUSCLE. Protein structure prediction was done using AlphaFold2 with ColabFold (45, 46).

## SUPPLEMENTAL MATERIAL

Fig. S1 mbio.01990-23-s0001.tif ΦE72 prevents growth of *S. epidermidis* 1457 wild type.

Fig S2 mbio.01990-23-s0002.tif Area-under-the-curve quantification of GroP-GroP-Glc.

Fig S3 mbio.01990-23-s0003.tif Biofilm formation.

Fig S4 mbio.01990-23-s0004.tif Electron microscopy.

Fig. S5 mbio.01990-23-s0005.tif Saturation of ΦE72 with purified WTA.

Fig. S6 mbio.01990-23-s0006.tif Structural prediction of the *S. epidermidis* TagE trimer with Alphafold2.

Fig. S7 mbio.01990-23-s0007.tif NMR spectra recorded for WTA isolated from *S. epidermidis* wild type.

Tables S1 to S4 mbio.01990-23-s0008.docx Bacteriophages, primers, NMR chemical shifts, and *tagE* conservation in *S. epidermidis*.

Supplemental material mbio.01990-23-s0009.docx Legends for supplemental figures and extended descriptions of detailed methods.

ASM does not own the copyrights to Supplemental Material that may be linked to, or accessed through, an article. The authors have granted ASM a non-exclusive, world-wide license to publish the Supplemental Material files. Please contact the corresponding author directly for reuse.
