# Supplementary figures and images for: Wall teichoic acid substitution with glucose governs phage susceptibility of Staphylococcus epidermidis
Source: mBio. 2024 Mar 12;15(4):e01990-23. doi: 10.1128/mbio.01990-23 (PMC11005348; doi:10.1128/mbio.01990-23)

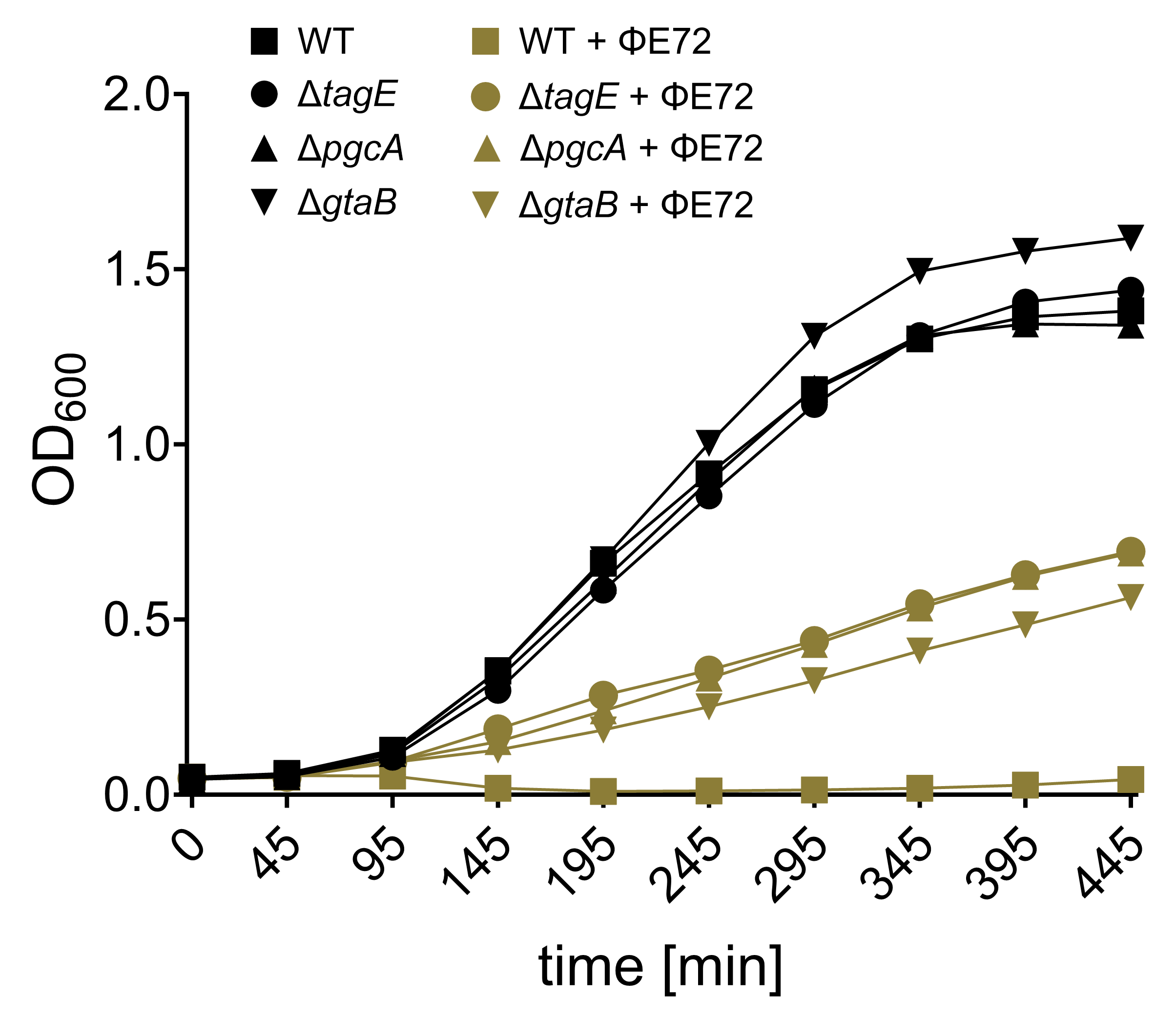

Supplement: Fig. S1 — ΦE72 prevents growth of S. epidermidis 1457 wild type. [file mbio.01990-23-s0001.tif]

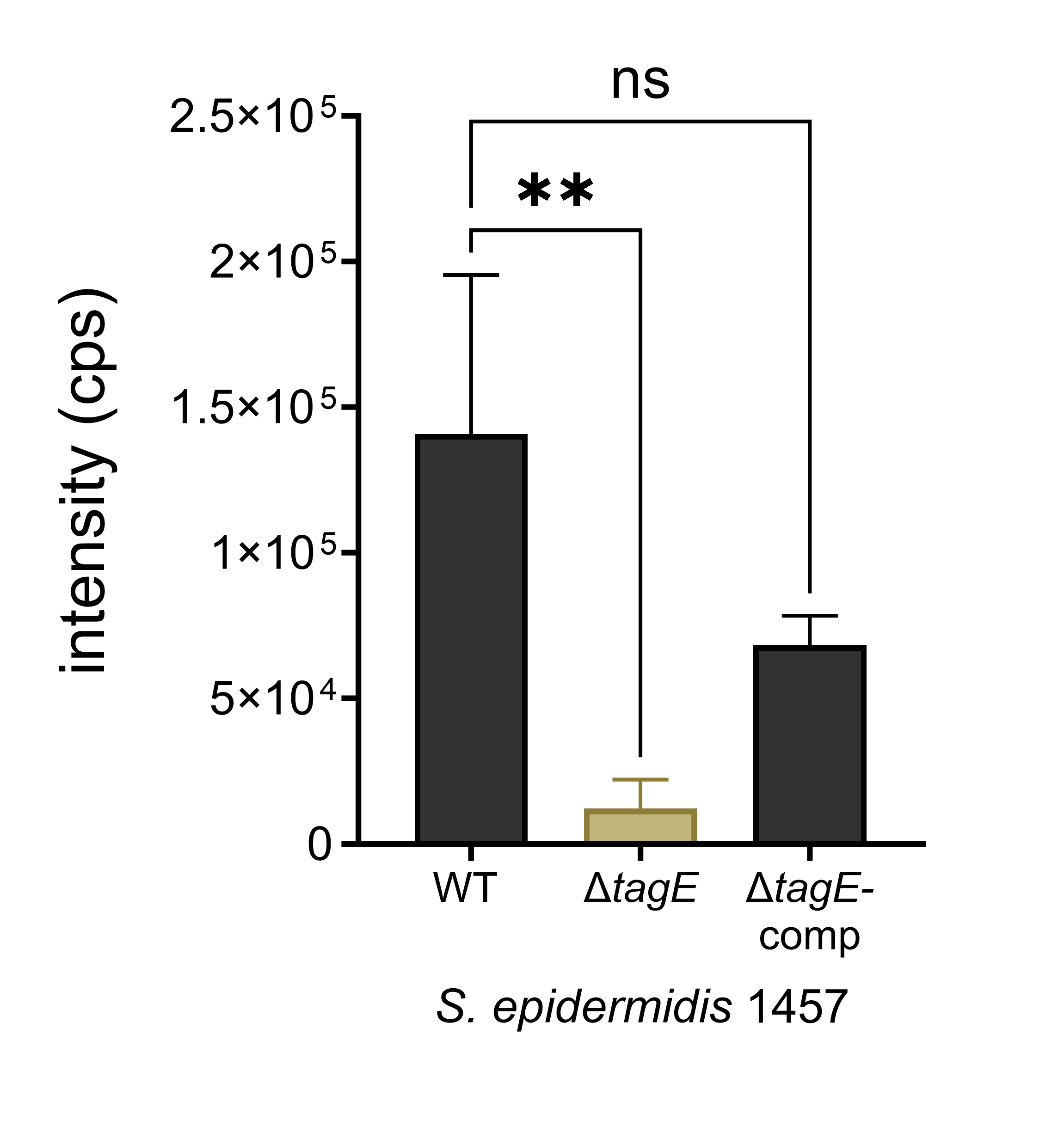

Supplement: Fig S2 — Area-under-the-curve quantification of GroP-GroP-Glc. [file mbio.01990-23-s0002.tif]

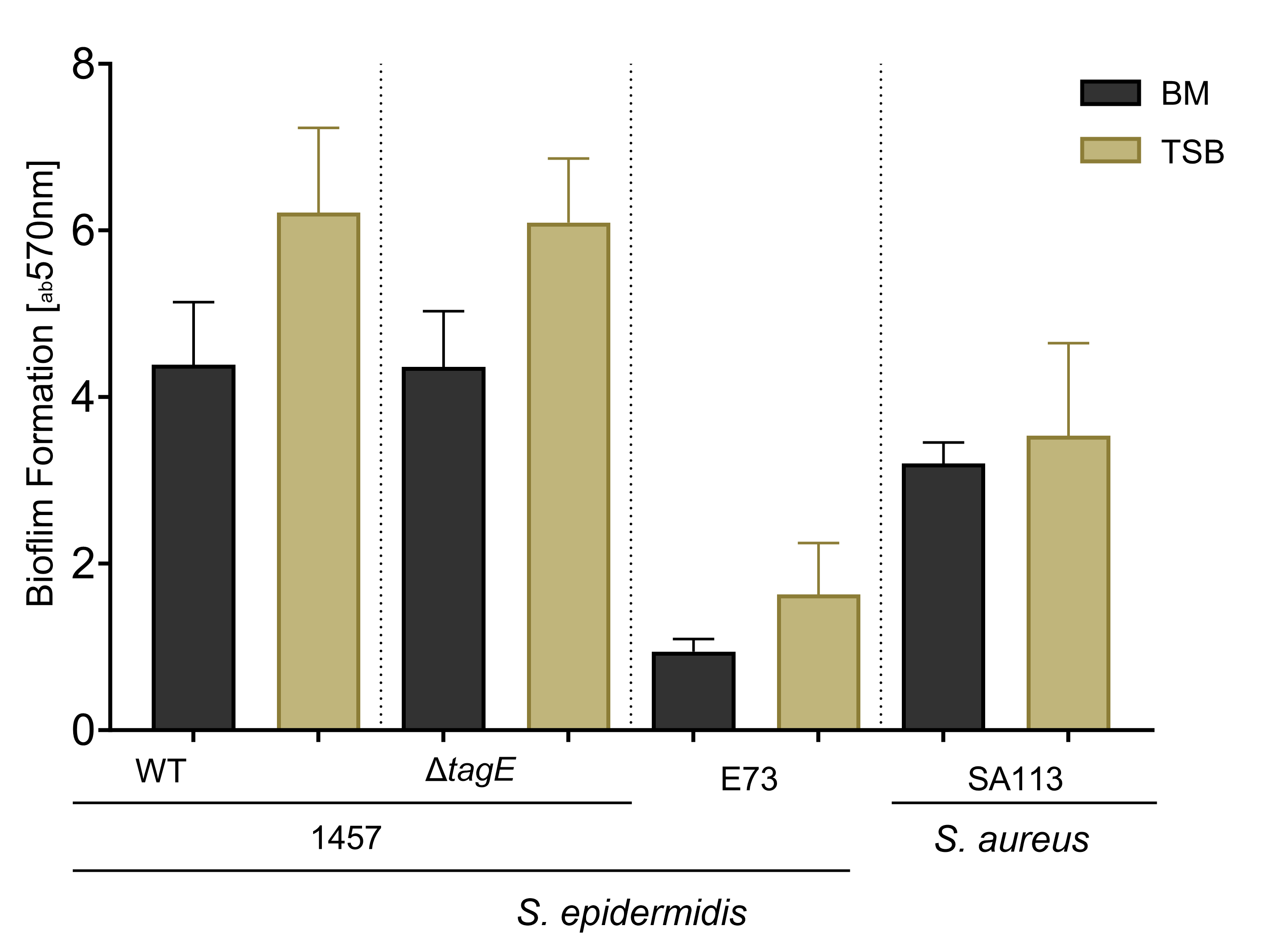

Supplement: Fig S3 — Biofilm formation. [file mbio.01990-23-s0003.tif]

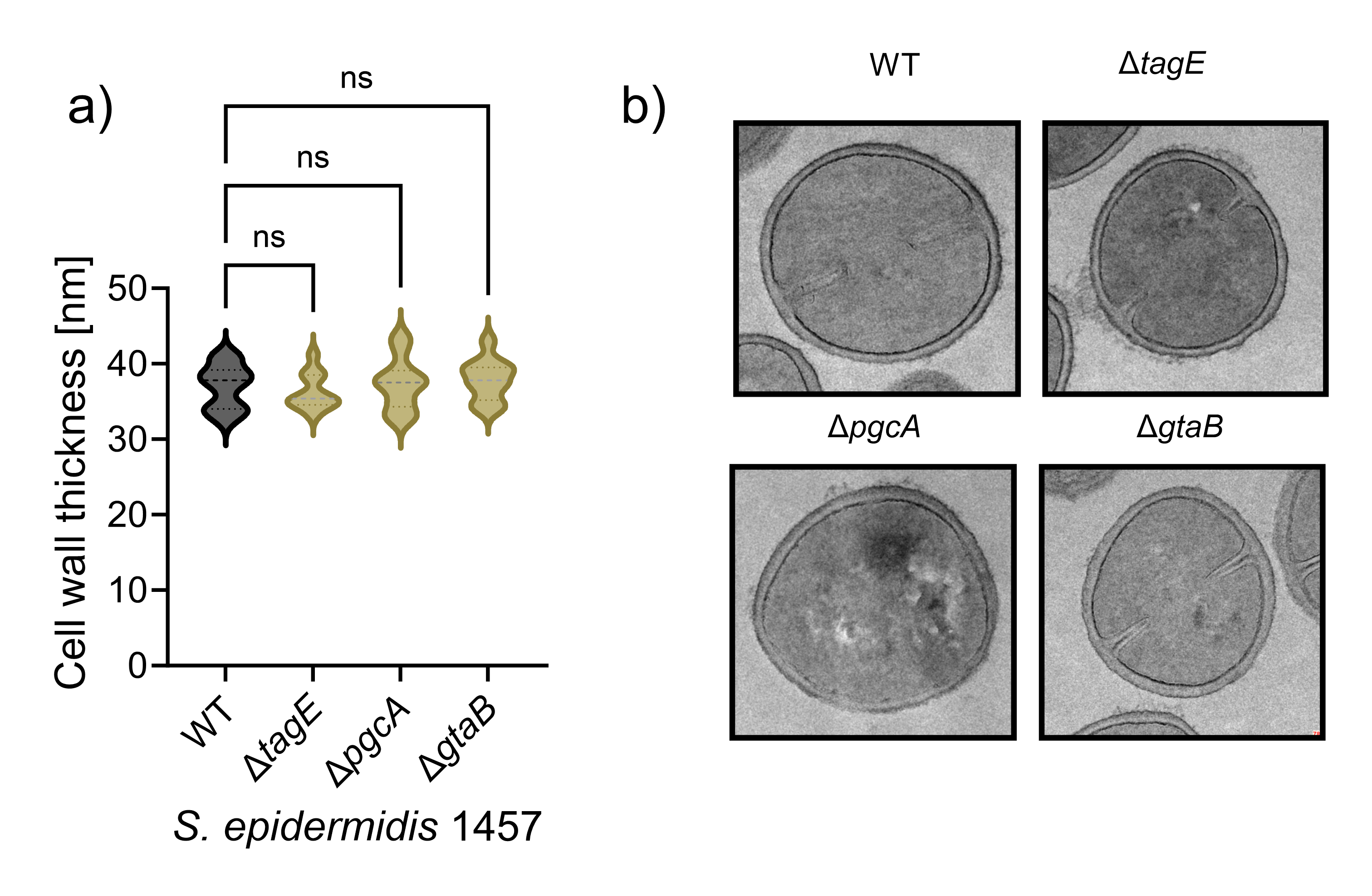

Supplement: Fig S4 — Electron microscopy. [file mbio.01990-23-s0004.tif]

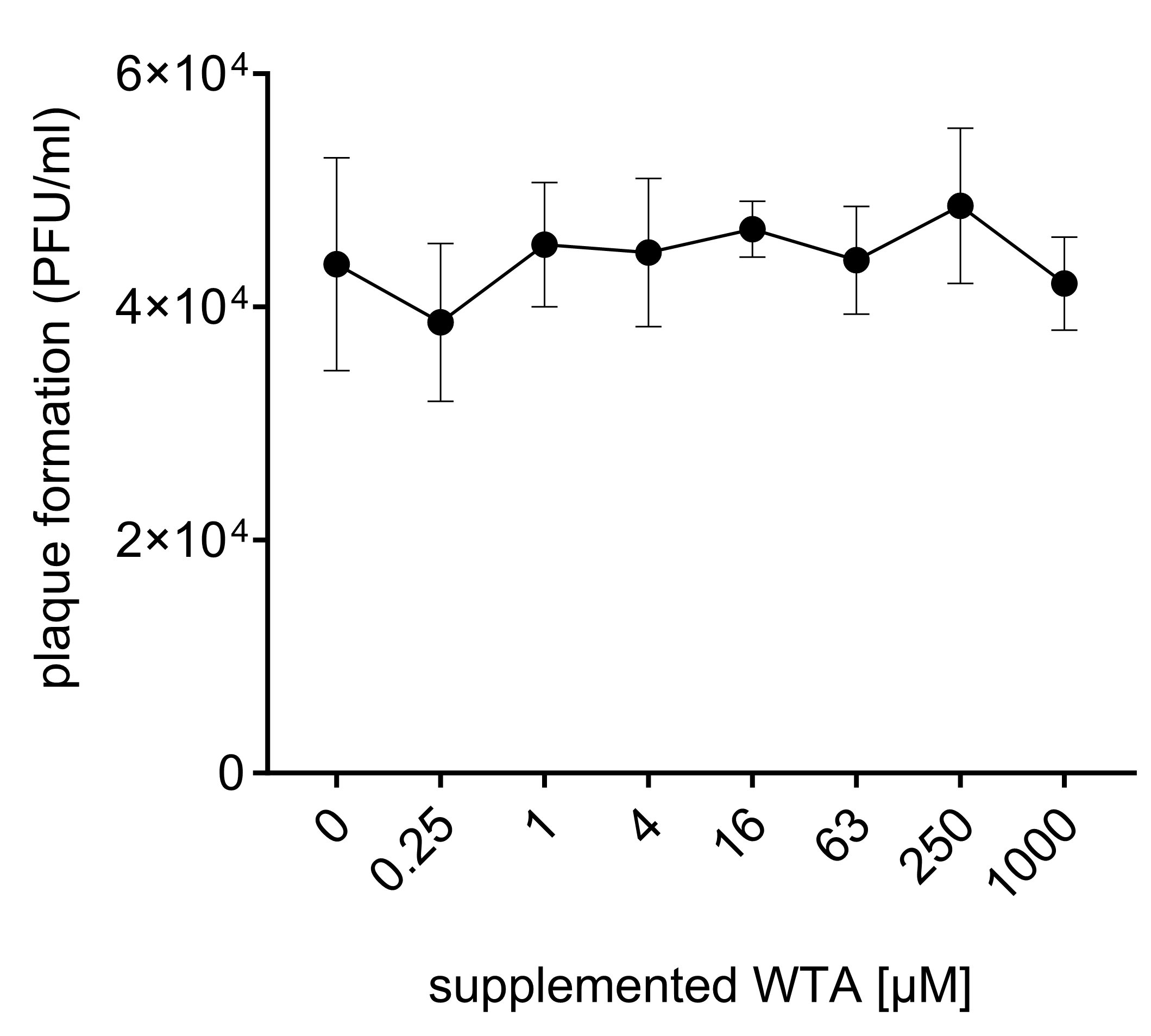

Supplement: Fig. S5 — Saturation of ΦE72 with purified WTA. [file mbio.01990-23-s0005.tif]

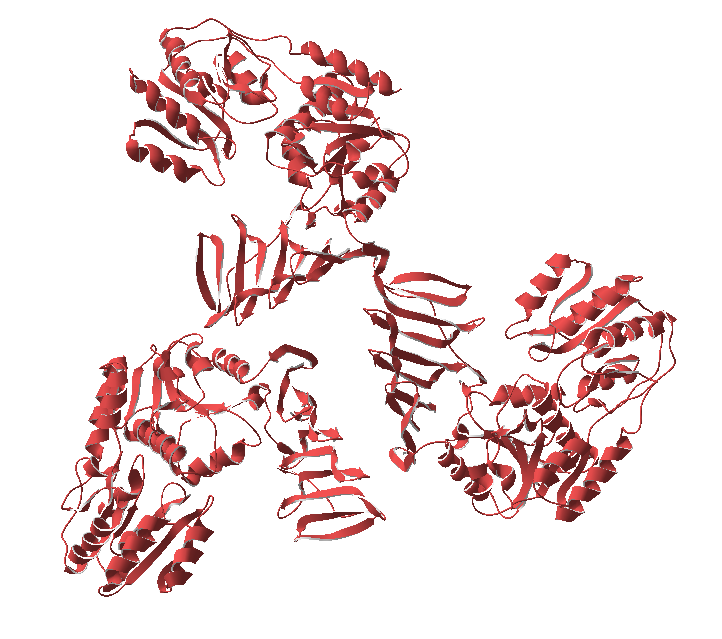

Supplement: Fig. S6 — Structural prediction of the S. epidermidis TagE trimer with Alphafold2. [file mbio.01990-23-s0006.tif]

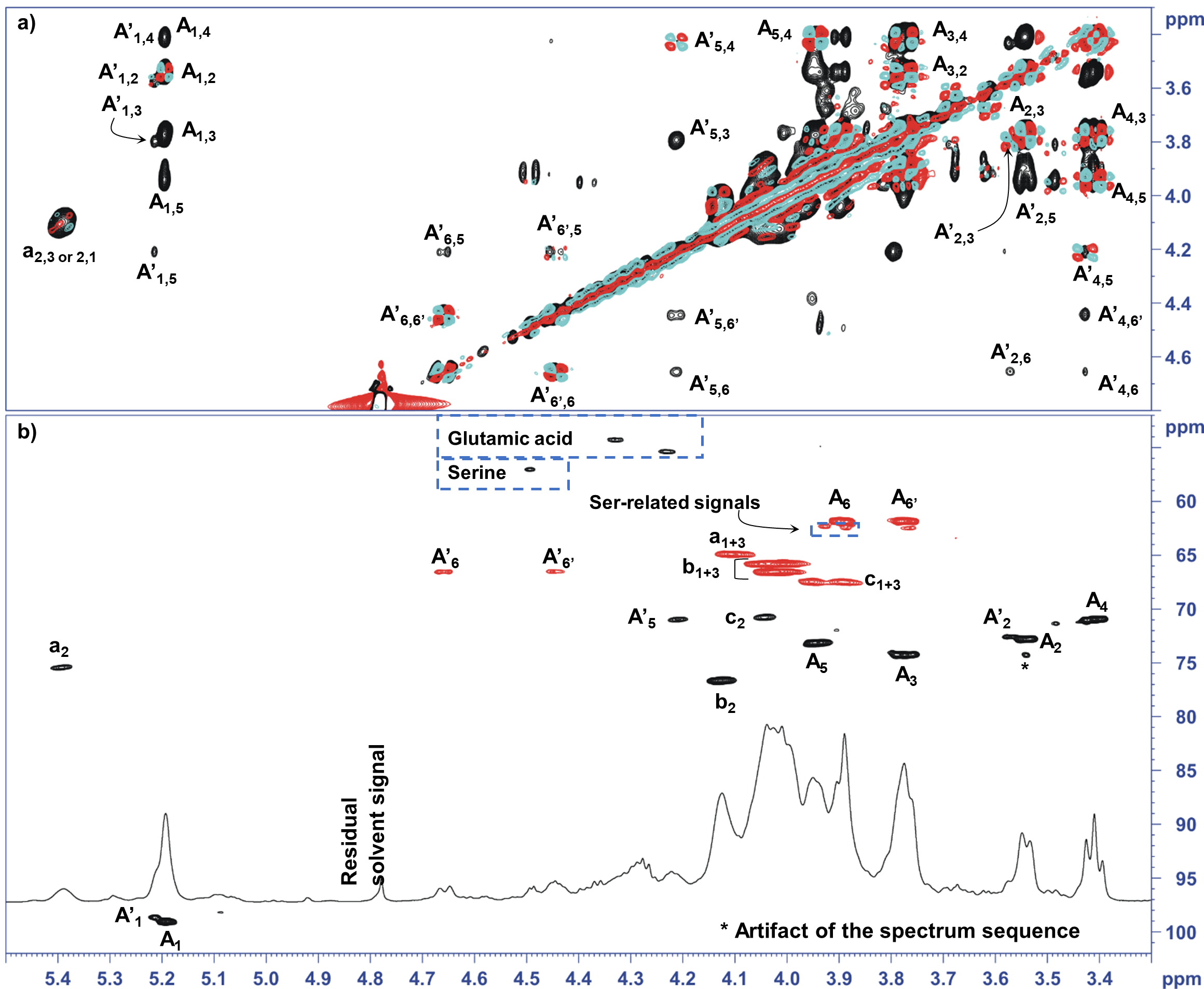

Supplement: Fig. S7 — NMR spectra recorded for WTA isolated from S. epidermidis wild type. [file mbio.01990-23-s0007.tif]
