# Supplementary material for: Wall teichoic acid substitution with glucose governs phage susceptibility of Staphylococcus epidermidis
Source: mBio. 2024 Mar 12;15(4):e01990-23. doi: 10.1128/mbio.01990-23 (PMC11005348; doi:10.1128/mbio.01990-23)
Supplement: Tables S1 to S4 — Bacteriophages, primers, NMR chemical shifts, and tagE conservation in S. epidermidis. [file mbio.01990-23-s0008.docx]

**Table S1:** Bacteriophages and bacterial strains used in this study.

| **Bacteriophage** | **Propagation strain** | **Morphology** | **Reference or origin** |
| --- | --- | --- | --- |
| ΦE72 | *S. epidermidis* 1457 | siphovirus | Fišarová et al |
| Φ459 | *S. epidermidis* SE459 | siphovirus | Fišarová et al |
| Φ456 | *S. epidermidis* SE456 | siphovirus | Fišarová et al |
| Φ27 | *S. epidermidis* SE27 | siphovirus | Fišarová et al |
| Φ15 | *S. epidermidis* SE15 | siphovirus | Fišarová et al |
| Φ187 | *S. aureus* PS187 | siphovirus | Pantůcek et al |
| ΦUKE3 | *S. epidermidis* DSM18857 | podovirus | DSMZ* |
| ΦSpree | *S. epidermidis* DSM18857 | podovirus | DSMZ* |
| ΦBE03 | *S. epidermidis* SKNA73 | podovirus | Valente et al.** |
| ΦBVG | *S. epidermidis* DSM20608 | podovirus | DSMZ* |
| ΦPauli | *S. epidermidis* DSM20608 | podovirus | DSMZ* |
| ΦNepomuk | *S. epidermidis* DSM20044 | podovirus | DSMZ* |
| ΦBE04 | *S. epidermidis* SKNA34 | myovirus | Valente et al.** |
| ΦBE06 | *S. epidermidis* SKNA34 | myovirus | Valente et al.** |
| ΦAlex | *S. epidermidis* DSM3269 | myovirus | DSMZ* |
| ΦK | *S. epidermidis* RN4220 | myovirus | O'Flaherty et al. |

* DSMZ: German Collection of Microorganisms and Cell Cultures

** Department of Intensive Care Medicine, Inselspital, Bern University Hospital, Switzerland

**Table S2:** Primer sequences used for cloning and sequencing.

| **Primer name** | **Primer sequence** | **Application** |
| --- | --- | --- |
| tagE F1-For | ATCTGAATTCAGTAAATCAGCATCAATAAG | Deletion of *S. epidermidis* *tagE* |
| tagE F1-Rev | TTTGAGATCTGAAATTTTATAATGTGATTTAAGAAG | Deletion of *S. epidermidis* *tagE* |
| tagE F2-For | TATAAGATCTTAGGTATTCAGATGGTTTAGATGATC | Deletion of *S. epidermidis* *tagE* |
| tagE F2-Rev | ATTAGTCGACAATGCATTAGAAGTTAAATTCGAAC | Deletion of *S. epidermidis* *tagE* |
| pgcA F1-For | AAGGGAATTCCAAAAGAAATGTTACCAATATTAG | Deletion of *S. epidermidis* *pgcA* |
| pgcA F1-Rev | TTTGAGATCTTAATATCGAAATAGAATTAACATG | Deletion of *S. epidermidis* *pgcA* |
| pgcA F2-For | TACGAGATCTTTCGAAAACATAAAAAGTTCTTAG | Deletion of *S. epidermidis* *pgcA* |
| pgcA F2-Rev | TTTTGTCGACTTGAATGAAATCTAATTCATTTGC | Deletion of *S. epidermidis* *pgcA* |
| gtaB F1-For | GTCTTGGAATTCTAATACCACTCGTATTTACAG | Deletion of *S. epidermidis* *gtaB* |
| gtaB F1-Rev | ATATAAGATCTACAGACATCCACTGAAAAACACTAG | Deletion of *S. epidermidis* *gtaB* |
| gtaB F2-For | GTCAAGATCTTTTGATTATTAGAAAGGATAGTACCC | Deletion of *S. epidermidis* *gtaB* |
| gtaB F2-Rev | TATCTGTCGACAACTTTAATCAATTTGAGTTAGTTG | Deletion of *S. epidermidis* *gtaB* |
| TagE Locus Comp-For | TCATGGTACCTTACTTTACTCTCTCAAACAAC | Fragment synthesis for complementation of gene locus containing *tagE*, *pgcA,* and *gtaB* |
| TagE Locus Comp-Rev | TTCTGTCGACATTCTTGATTAAGTTAATGTTAATATTG | Fragment synthesis for complementation of gene locus containing *tagE*, *pgcA,* and *gtaB* |
| 473 Eco | CCTCAAGCTAGAGAGTCATTACCCC | sequencing of pRB473 and pBASE shuttle vectors |
| 473 Hind | CTGGATTTGTTCAGAACGCTCGG | sequencing of pRB473 shuttle vector |
| pBASE Hind | CTACTTCTTTCAAACTCTCTCTACG | sequencing of pBase shuttle vector |
| erm-For | CTATTATTTAACGGGAGGAAA | Sequencing from erythromycin cassette forward |
| erm-Rev | TAATCTAACGTATTTATCTGCGTA | Sequencing from erythromycin cassette reverse |

**Table S3**: NMR chemical shifts. ^1^H (600MHz) chemical shifts of WTA structural motifs found in *S. epidermidis* wild type. The sample was dissolved in deuterated water (HOD, 550 μl) and measured at 298 K. By convention, C-1 of the glycerol unit is placed at the left of the structural formula, P stands for phosphate.

| **Residue** | **motif** | **1;1’ (for Gro)** | **2** | **3; 3’ (for Gro)** | **4** | **5** | **6; 6’** |
| --- | --- | --- | --- | --- | --- | --- | --- |
| **a** |  | 4.11 x 2 | 5.39 | 4.11 x 2 | -- | -- | -- |
| **Gro** |  | 64.9 | 75.5 | 64.9 | -- | -- | -- |
| **b** |  | ~ 4.02 x 2* | 4.12 | ~ 4.05 – 4.00* | -- | -- | -- |
| **Gro** |  | 66.6 | 76.7 | 65.8 | -- | -- | -- |
| **c** |  | 3.85;3.90 | 4.04 | 3.85;3.90 | -- | -- | -- |
| **Gro** |  | 67.5 | 70.8 | 67.5 | -- | -- | -- |
| **A** |  | 5.20 | 3.54 | 3.78 | 3.41 | 3.95 | 3.89; 3.77 |
| **t-α-Glc** |  | 98.9 | 72.8 | 74.3 | 71.0 | 73.1 | 61.9 |
| **A’** |  | 5.22 | 3.57 | 3.80 | 3.43 | 4.21 | 4.66; 4.44 |
| **t-α-Glc6Ala** |  | 96.2 | 72.6 | 74.1 | 71.2 | 71.0 | 66.5 |
| **Ala** |  | -- | 4.23 | 1.62 | -- | -- | -- |
|  |  | 171.5 | 50.1 | 16.6-- | -- | -- | -- |

* These signals can be exchanged.

**Table S4:** The nucleotide sequence of *S. epidermidis* 1457 *tagE* was blasted against all 218 *S. epidermidis* genomes available at the NCBI genome database. Nucleotide conservation, query coverage and flanking base pairs are shown.

| **Accession-Nr. of *S. epidermidis* genome** | **Nucleotide sequence conservation [%]** | **Query coverage [bp]** | **Flanking base pairs** | |
| --- | --- | --- | --- | --- |
| LR735432.1 | 100.000 | 1491 | 540407 | 538917 |
| CP121509.1 | 100.000 | 1491 | 623201 | 621711 |
| CP094728.1 | 100.000 | 1491 | 517857 | 516367 |
| CP090575.1 | 100.000 | 1491 | 444950 | 443460 |
| CP070057.1 | 100.000 | 1491 | 1234798 | 1236288 |
| CP043781.1 | 100.000 | 1491 | 802308 | 800818 |
| CP020463.1 | 100.000 | 1491 | 477023 | 475533 |
| CP000029.1 | 100.000 | 1491 | 2077256 | 2078746 |
| CP133254.1 | 99.531 | 1491 | 186275 | 187765 |
| LR735434.1 | 98.860 | 1491 | 480695 | 479205 |
| LR134536.1 | 98.860 | 1491 | 465413 | 463923 |
| CP133663.1 | 98.860 | 1491 | 511762 | 510272 |
| CP120426.1 | 98.860 | 1491 | 367868 | 369358 |
| CP120425.1 | 98.860 | 1491 | 1852432 | 1850942 |
| CP120423.1 | 98.860 | 1491 | 368103 | 369593 |
| CP094733.1 | 98.860 | 1491 | 532648 | 531158 |
| CP094726.1 | 98.860 | 1491 | 531345 | 529855 |
| CP121231.1 | 98.860 | 1491 | 2246656 | 2245166 |
| CP102565.2 | 98.860 | 1491 | 458174 | 456684 |
| CP093212.1 | 98.860 | 1491 | 519324 | 517834 |
| CP093198.1 | 98.860 | 1491 | 416997 | 415507 |
| CP093196.1 | 98.860 | 1491 | 520898 | 519408 |
| CP093181.1 | 98.860 | 1491 | 2239113 | 2240603 |
| CP093179.1 | 98.860 | 1491 | 2283169 | 2284659 |
| CP097514.1 | 98.860 | 1491 | 471552 | 470062 |
| CP097512.1 | 98.860 | 1491 | 471552 | 470062 |
| CP073878.1 | 98.860 | 1491 | 2170524 | 2172014 |
| CP073904.1 | 98.860 | 1491 | 2170541 | 2172031 |
| CP073900.1 | 98.860 | 1491 | 2170561 | 2172051 |
| CP073898.1 | 98.860 | 1491 | 609935 | 608445 |
| CP073895.1 | 98.860 | 1491 | 2169265 | 2170755 |
| CP073893.1 | 98.860 | 1491 | 545380 | 543890 |
| CP073890.1 | 98.860 | 1491 | 2170556 | 2172046 |
| CP073887.1 | 98.860 | 1491 | 2169408 | 2170898 |
| CP073883.1 | 98.860 | 1491 | 1883640 | 1885130 |
| CP073881.1 | 98.860 | 1491 | 2170456 | 2171946 |
| CP073876.1 | 98.860 | 1491 | 542775 | 541285 |
| CP073872.1 | 98.860 | 1491 | 545404 | 543914 |
| CP073869.1 | 98.860 | 1491 | 545240 | 543750 |
| CP073865.1 | 98.860 | 1491 | 2420501 | 2421991 |
| CP073863.1 | 98.860 | 1491 | 545399 | 543909 |
| CP073862.1 | 98.860 | 1491 | 542883 | 541393 |
| CP073859.1 | 98.860 | 1491 | 545399 | 543909 |
| CP073857.1 | 98.860 | 1491 | 542903 | 541413 |
| CP073855.1 | 98.860 | 1491 | 542979 | 541489 |
| CP073852.1 | 98.860 | 1491 | 2175243 | 2176733 |
| CP073850.1 | 98.860 | 1491 | 2170549 | 2172039 |
| CP073847.1 | 98.860 | 1491 | 543953 | 542463 |
| CP073844.1 | 98.860 | 1491 | 2170465 | 2171955 |
| CP073841.1 | 98.860 | 1491 | 2170495 | 2171985 |
| CP073840.1 | 98.860 | 1491 | 2169429 | 2170919 |
| CP073836.1 | 98.860 | 1491 | 2170657 | 2172147 |
| CP073835.1 | 98.860 | 1491 | 540648 | 539158 |
| CP073830.1 | 98.860 | 1491 | 543954 | 542464 |
| CP073827.1 | 98.860 | 1491 | 543927 | 542437 |
| CP073821.1 | 98.860 | 1491 | 2170556 | 2172046 |
| CP073824.1 | 98.860 | 1491 | 2168005 | 2169495 |
| CP060157.1 | 98.860 | 1491 | 1912432 | 1910942 |
| CP060256.1 | 98.860 | 1491 | 622398 | 620908 |
| CP060258.1 | 98.860 | 1491 | 567468 | 565978 |
| CP060255.1 | 98.860 | 1491 | 483191 | 481701 |
| CP060254.1 | 98.860 | 1491 | 482439 | 480949 |
| CP060253.1 | 98.860 | 1491 | 482133 | 480643 |
| CP060252.1 | 98.860 | 1491 | 622646 | 621156 |
| CP060251.1 | 98.860 | 1491 | 429012 | 427522 |
| CP052994.1 | 98.860 | 1491 | 513240 | 511750 |
| CP052990.1 | 98.860 | 1491 | 550749 | 549259 |
| CP052984.1 | 98.860 | 1491 | 474995 | 473505 |
| CP052981.1 | 98.860 | 1491 | 459147 | 457657 |
| CP053088.1 | 98.860 | 1491 | 502855 | 501365 |
| CP052959.1 | 98.860 | 1491 | 506343 | 504853 |
| CP052955.1 | 98.860 | 1491 | 561991 | 560501 |
| CP052940.1 | 98.860 | 1491 | 459074 | 457584 |
| CP052939.1 | 98.860 | 1491 | 549065 | 547575 |
| CP066303.1 | 98.860 | 1491 | 1993366 | 1994856 |
| CP040883.1 | 98.860 | 1491 | 479859 | 478369 |
| CP045648.1 | 98.860 | 1491 | 515508 | 514018 |
| CP013943.1 | 98.860 | 1491 | 2190329 | 2191819 |
| LT571449.1 | 98.793 | 1491 | 2290418 | 2291908 |
| CP121522.1 | 98.793 | 1491 | 624979 | 623489 |
| CP120424.1 | 98.793 | 1491 | 603000 | 601510 |
| CP090989.1 | 98.793 | 1491 | 515939 | 514449 |
| CP060247.1 | 98.793 | 1491 | 532971 | 531481 |
| CP052967.1 | 98.793 | 1491 | 517987 | 516497 |
| CP030246.1 | 98.793 | 1491 | 504578 | 503088 |
| CP121483.1 | 98.726 | 1491 | 2125053 | 2123563 |
| CP090922.1 | 98.592 | 1491 | 465646 | 464156 |
| CP127869.1 | 98.524 | 1491 | 496817 | 495327 |
| CP118842.1 | 98.524 | 1491 | 497761 | 496271 |
| CP121527.1 | 98.524 | 1491 | 2202909 | 2201419 |
| CP064359.1 | 98.524 | 1491 | 2063098 | 2064588 |
| CP094872.1 | 98.524 | 1491 | 520067 | 518577 |
| CP094865.1 | 98.524 | 1491 | 520020 | 518530 |
| CP094859.1 | 98.524 | 1491 | 520063 | 518573 |
| CP093222.1 | 98.524 | 1491 | 428939 | 427449 |
| CP069951.1 | 98.524 | 1491 | 1934592 | 1936082 |
| CP069473.1 | 98.524 | 1491 | 1056238 | 1054748 |
| CP065656.1 | 98.524 | 1491 | 113856 | 112366 |
| CP043796.1 | 98.524 | 1491 | 260874 | 259384 |
| CP043777.1 | 98.524 | 1491 | 1274600 | 1273110 |
| CP043845.1 | 98.524 | 1491 | 170944 | 172434 |
| CP028282.1 | 98.524 | 1491 | 2082686 | 2084176 |
| AE015929.1 | 98.524 | 1491 | 2082501 | 2083991 |
| LR735440.1 | 98.457 | 1491 | 549708 | 548218 |
| CP093208.1 | 98.457 | 1491 | 499826 | 498336 |
| CP090915.1 | 98.457 | 1491 | 460709 | 459219 |
| CP052948.1 | 98.457 | 1491 | 502269 | 500779 |
| CP069219.1 | 98.457 | 1491 | 534530 | 533040 |
| CP066376.1 | 98.457 | 1491 | 467125 | 465635 |
| CP129365.1 | 98.390 | 1491 | 473643 | 472153 |
| CP118829.1 | 98.390 | 1491 | 524530 | 523040 |
| CP118781.1 | 98.390 | 1491 | 524535 | 523045 |
| CP121513.1 | 98.390 | 1491 | 640336 | 638846 |
| CP121518.1 | 98.390 | 1491 | 361643 | 363133 |
| CP121480.1 | 98.390 | 1491 | 2116206 | 2114716 |
| CP121477.1 | 98.390 | 1491 | 2142535 | 2141045 |
| CP009046.1 | 98.390 | 1491 | 1002860 | 1004350 |
| CP082816.1 | 98.390 | 1491 | 512418 | 510929 |
| CP118834.1 | 98.323 | 1491 | 524577 | 523087 |
| CP110611.1 | 98.256 | 1491 | 466181 | 464691 |
| HG813242.1 | 98.256 | 1491 | 2085109 | 2086599 |
| CP101316.1 | 98.125 | 1493 | 1470070 | 1471562 |
| CP119047.1 | 98.122 | 1491 | 475621 | 474131 |
| CP060528.1 | 98.122 | 1491 | 475188 | 473698 |
| CP090941.1 | 97.985 | 1489 | 464773 | 463285 |
| CP090924.1 | 97.924 | 1493 | 249305 | 247815 |
| CP121486.1 | 97.857 | 1493 | 484579 | 486069 |
| CP129374.1 | 97.723 | 1493 | 486298 | 484808 |
| CP094731.1 | 97.723 | 1493 | 532572 | 531082 |
| CP094729.1 | 97.723 | 1493 | 507481 | 505991 |
| CP093188.1 | 97.723 | 1493 | 534368 | 532878 |
| CP071992.1 | 97.723 | 1493 | 512955 | 511465 |
| CP043801.1 | 97.720 | 1491 | 2363240 | 2364730 |
| CP043792.1 | 97.720 | 1491 | 1101512 | 1100022 |
| CP034115.1 | 97.723 | 1493 | 1173954 | 1172464 |
| CP034111.1 | 97.723 | 1493 | 1134225 | 1132735 |
| LR735437.1 | 97.653 | 1491 | 572205 | 570715 |
| LR735421.1 | 97.653 | 1491 | 492909 | 491419 |
| CP118814.1 | 97.653 | 1491 | 497579 | 496089 |
| CP121529.1 | 97.653 | 1491 | 650183 | 648693 |
| CP121502.1 | 97.653 | 1491 | 1777971 | 1779461 |
| CP121497.1 | 97.653 | 1491 | 628679 | 627189 |
| CP121526.1 | 97.653 | 1491 | 1838032 | 1839522 |
| CP121507.1 | 97.653 | 1491 | 737072 | 735582 |
| CP120427.1 | 97.653 | 1491 | 1548019 | 1546529 |
| CP111111.1 | 97.653 | 1491 | 482189 | 480699 |
| CP100383.1 | 97.653 | 1491 | 501325 | 502815 |
| CP093210.1 | 97.653 | 1491 | 1994438 | 1995928 |
| CP093205.1 | 97.653 | 1491 | 562557 | 561067 |
| CP093202.1 | 97.653 | 1491 | 491143 | 489653 |
| CP093193.1 | 97.653 | 1491 | 510977 | 509487 |
| CP093173.1 | 97.653 | 1491 | 530898 | 529408 |
| CP093165.1 | 97.653 | 1491 | 477581 | 476091 |
| CP097519.1 | 97.653 | 1491 | 446521 | 445031 |
| CP095090.1 | 97.653 | 1491 | 496740 | 495250 |
| CP090912.1 | 97.656 | 1493 | 430416 | 428926 |
| CP071988.1 | 97.653 | 1491 | 493973 | 492483 |
| CP084008.1 | 97.653 | 1491 | 2326946 | 2328436 |
| CP060257.1 | 97.653 | 1491 | 495639 | 494149 |
| CP060249.1 | 97.653 | 1491 | 473760 | 472270 |
| CP060248.1 | 97.653 | 1491 | 470870 | 469380 |
| CP053007.1 | 97.656 | 1493 | 498450 | 496960 |
| CP052975.1 | 97.653 | 1491 | 2071263 | 2072753 |
| CP052971.1 | 97.653 | 1491 | 1956229 | 1957719 |
| CP060250.1 | 97.653 | 1491 | 427457 | 425967 |
| CP052998.1 | 97.653 | 1491 | 2101581 | 2103071 |
| CP052997.1 | 97.653 | 1491 | 486761 | 485271 |
| CP052991.1 | 97.653 | 1491 | 2087909 | 2089399 |
| CP052978.1 | 97.653 | 1491 | 2018039 | 2019529 |
| CP052969.1 | 97.653 | 1491 | 519647 | 518157 |
| CP052963.1 | 97.653 | 1491 | 532417 | 530927 |
| CP052962.1 | 97.653 | 1491 | 529789 | 528299 |
| CP052953.1 | 97.653 | 1491 | 511084 | 509594 |
| CP052951.1 | 97.653 | 1491 | 2211729 | 2213219 |
| CP052985.1 | 97.653 | 1491 | 2139853 | 2141343 |
| CP069215.1 | 97.656 | 1493 | 469328 | 467838 |
| CP060794.1 | 97.653 | 1491 | 269352 | 270842 |
| CP043804.1 | 97.656 | 1493 | 512245 | 513735 |
| CP035643.1 | 97.653 | 1491 | 2014552 | 2016042 |
| CP040864.1 | 97.653 | 1491 | 370830 | 369340 |
| CP040867.1 | 97.653 | 1491 | 472208 | 470718 |
| CP040868.1 | 97.653 | 1491 | 462570 | 461080 |
| CP014119.1 | 97.653 | 1491 | 518796 | 520286 |
| CP024408.1 | 97.656 | 1493 | 83951 | 82462 |
| CP102567.2 | 97.586 | 1491 | 1843270 | 1844760 |
| CP093185.1 | 97.586 | 1491 | 528346 | 526856 |
| CP071996.1 | 97.589 | 1493 | 2097736 | 2096246 |
| CP052973.1 | 97.586 | 1491 | 563799 | 562309 |
| CP068136.1 | 97.589 | 1493 | 822754 | 821264 |
| CP014132.1 | 97.586 | 1491 | 2404226 | 2405716 |
| CP043788.1 | 97.648 | 1488 | 1888299 | 1889784 |
| CP024437.1 | 97.773 | 1482 | 91242 | 89763 |
| CP118820.1 | 97.522 | 1493 | 538089 | 536599 |
| CP093170.1 | 97.522 | 1493 | 536830 | 535340 |
| CP043784.1 | 97.522 | 1493 | 1438876 | 1437386 |
| CP061029.1 | 97.706 | 1482 | 1819863 | 1821342 |
| CP069954.1 | 97.388 | 1493 | 854965 | 856455 |
| AP019721.1 | 97.388 | 1493 | 491081 | 489591 |
| CP035288.1 | 97.388 | 1493 | 536665 | 535175 |
| CP010942.1 | 97.386 | 1492 | 1876621 | 1878110 |
| CP129372.1 | 97.571 | 1482 | 486085 | 484606 |
| CP052941.1 | 97.571 | 1482 | 489816 | 488337 |
| CP053002.1 | 97.323 | 1494 | 523613 | 522122 |
| CP053005.1 | 97.254 | 1493 | 478971 | 477481 |
| LR735429.1 | 97.187 | 1493 | 501753 | 500263 |
| CP053000.1 | 97.187 | 1493 | 501180 | 499690 |
| CP033782.1 | 97.187 | 1493 | 447225 | 448715 |
| CP106834.1 | 97.049 | 1491 | 450817 | 449339 |
| CP090993.1 | 97.049 | 1491 | 443740 | 442262 |
| CP090985.1 | 97.049 | 1491 | 1995217 | 1996695 |
| CP091006.1 | 97.049 | 1491 | 443737 | 442259 |
| CP071994.1 | 97.049 | 1491 | 461430 | 459952 |
| CP043841.1 | 97.813 | 1143 | 971771 | 972913 |
| CP043841.1 | 97.199 | 357 | 1782157 | 1781801 |
| CP088002.1 | 98.969 | 776 | 1997353 | 1998128 |
| CP043847.1 | 98.325 | 776 | 1990890 | 1990115 |
| CP090998.1 | 98.325 | 776 | 453604 | 452836 |
| CP018842.1 | 96.658 | 778 | 152937 | 152162 |
| CP022247.1 | 96.401 | 778 | 2398028 | 2397253 |
