# Supplementary material for: Wall teichoic acid substitution with glucose governs phage susceptibility of Staphylococcus epidermidis
Source: mBio. 2024 Mar 12;15(4):e01990-23. doi: 10.1128/mbio.01990-23 (PMC11005348; doi:10.1128/mbio.01990-23)
Supplement: Supplemental material — Legends for supplemental figures and extended descriptions of detailed methods. [file mbio.01990-23-s0009.docx]

**Fig. S1:** ΦE72 prevents growth of *S. epidermidis* 1457 wild type (WT). Growth of the Δ*tagE*, Δ*pgcA,* Δ*gtaB* mutants is only partially reduced by ΦE72 compared to growth without addition of phage. Approximately 5x10^8^ PFU/ml were used. Data represent mean ± SEM of three independent experiments.

**Fig. S2:** Area-under-the-curve quantification of GroP-GroP-Glc residue ([M - H]^-^ = 487.0623) total ion current (TIC) chromatogram measured by HPLC-MS after chemical digest of *S. epidermidis* WTA. Data represent mean ± SEM of three independent experiments. Ordinary one-way ANOVA was used to determine statistical significance versus *S. epidermidis* 1457 wild type (WT), followed by Dunnett’s multiple comparisons tests, indicated as: not significant (ns), **P < 0.01.

**Fig. S3:** *S. epidermidis* 1457 biofilm formation was measured in BM and TSB medium. Biofilm formation is unchanged in the ∆*tagE* deletion mutant.

**Fig. S4:** Electron microscopy at 12,500 x magnification indicates that cell wall thickness (a), and cell shape (b), is unchanged in all mutants compared to the wild type. a) shows the mean cell wall thickness of at least 11 different bacterial cells of each mutant or the wild type (WT). Ordinary one-way ANOVA was used to determine statistical significance versus *S. epidermidis* 1457 wild type (WT), followed by Dunnett’s multiple comparisons tests, indicated as: not significant (ns).

**Fig. S5:** Saturation of ΦE72 with purified WTA isolated from *S. epidermidis* 1457 wild type.

**Fig. S6:** Structural prediction of the *S. epidermidis* TagE trimer with Alphafold2 (45, 46).

**Fig. S7:** NMR spectra recorded for WTA isolated from *S. epidermidis* wild type. a) Expansion of the HSQC spectrum detailing the anomeric and the carbinolic region. b) Overlap of the TOCSY (black) and COSY (cyan and red) spectra. In all the spectra, the most relevant densities are labelled with the letter used in Table S3; as for the carbohydrate units (**A** and **A’**), the anomeric signals are indicated with a capital letter, while the Gro units (**a**, **b**, and **c**) are labeled with small letters.

Extended descriptions of detailed methods

**WTA compositional analysis**

**HPLC-MS**

Analysis of the WTA polymer composition was performed using an LTQ Orbitrap Velos mass spectrometer (Thermo Fisher Scientific), connected to an ACQUITY ultra-performance liquid chromatography (UPLC) system (Waters Corporation). Separation in the UPLC was carried out using a Phenomenex C18-Gemini® column (150 × 2 mm, 3 μm, 110 Å, Phenomenex) at 37°C with 0.1% formic acid and 0.05% HCO_2_NH_4_ (A) and CH_3_CN (B) buffer system. A single run (injection volume of 5 μl) was performed with a flow rate of 0.2 ml/min and a two-step gradient: after 2.5 min of equilibration with 100% A, a 1-min gradient up to 5% B was followed by a 4-min gradient up to 70% B. After 2 min at 70% B, a re-equilibration step of 2.5 min followed with a flow rate of 4 ml/min. LC-MS data processing was done with UmetaFlow GUI ((57), https://github.com/axelwalter/streamlit-metabolomics-statistics) via extracted ion chromatograms with a mass tolerance of 10 ppm.

**NMR**

1H NMR spectra were recorded for both, wild-type and Δ*tagE* *S. epidermidis* strains, and they were carried out on a Bruker DRX-600 spectrometer equipped with a cryo-probe, at 298 K. Chemical shifts of spectra recorded in D_2_O were calculated in ppm relative to internal acetone (2.225 and 31.45 ppm). 2D NMR spectra were acquired for *S. epidermidis* wild type only, the spectral width was set to 12 ppm and the frequency carrier placed at the residual HOD peak, suppressed by pre-saturation. Two-dimensional spectra (DQ-COSY, TOCSY, NOESY, gHSQC, and gHMBC) were measured using standard Bruker software. For all experiments, 512 FIDs of 2,048 complex data points were collected, 32 scans per FID were acquired for homonuclear spectra, and 100 and 200 ms of mixing time was used for the TOCSY and NOESY spectra, respectively. Heteronuclear ^1^H-^13^C spectra were measured in the ^1^H-detected mode, gHSQC spectrum was acquired with 40 scans per FID, the GARP sequence was used for ^13^C decoupling during acquisition; gHMBC scans doubled those of gHSQC spectrum. During processing, each data matrix was zero-filled in both dimensions to give a matrix of 4K × 2K points and was resolution-enhanced in both dimensions by a cosine-bell function before Fourier transformation; data processing and analysis were performed with the Bruker Topspin 3 program.

**NMR analysis of the WTA of the wild type (WT) strain of *Staphylococcus epidermidis***

NMR analyses of the spectra displayed several signals in the anomeric region (5.5 – 4.4 ppm, Fig. 3c) of the proton spectrum with the one at 5.20 ppm being more intense than the others. Then, inspection of the HSQC spectrum (Fig S7a) disclosed that only the signals at 5.2 and 5.1 ppm arose by the anomeric position of different monosaccharide residues, due to the characteristic values of the related carbon atoms (Table S3, (58)). The full assignment of both proton and carbon chemical shifts was possible with confidence only for the most abundant unit, labelled with **A.** Thus, the anomeric proton at 5.2 ppm was labelled **A_1_**, and the combined analysis of the TOCSY and COSY spectra determined that it was an α-glucose (Fig. S7b). Indeed, the TOCSY spectrum showed that **A_1_** correlated to four other protons as occurs for *gluco* configured residues, and this information combined with those from the COSY spectrum enabled the sequence assignment from H-2 to H-5 (Fig. S7b, Table S3). Then, the identification of A6 was inferred by the finding of the H-4/H-6 cross peak in the TOCSY spectrum (Fig. S7b) while the position of the other H-6 proton, labelled A6’ was determined by the strong cross-peak in the COSY spectrum (Fig. S7b). Finally, the identification of the carbon chemical shifts was inferred by analysing the ^1^H-^13^C HSQC (Fig. S7a), which determined that **A** was a glucose unit that was not further substituted due to the similarity of its carbon chemical shifts to those reported for the reference glycoside (58). The inspection of the HMBC spectrum (not shown) reported a cross peak connecting H-1 of **A** to a carbon at 76.7 ppm in turn correlated to a proton at 4.12 ppm, later assigned to H-2/C-2 of a glycerol (Gro) unit, labelled **b**.

Interestingly, H-1 of **A** was flanked by a second anomeric proton at 5.22 ppm (Fig. S7b, Table S3), labelled as **A’** and presenting a correlation pattern in the TOCSY spectrum very similar to that of **A**, except for the fact that the density analogue to **A_1,5_** was missing while there was a new one relating H-1 to a proton at 4.21 ppm. The identification of the sequence between the protons of this second spin system was aided by the COSY spectrum and the additional signal at 4.21 was assigned to H-5, in turn correlated to the two H-6 protons at 4.66 and 4.44 ppm (Fig. S7b), highly deshielded due to the O-acylation with an Ala residue as inferred by the long range correlation with a carbonyl group at 171.5 ppm (not shown).

Then, the anomeric region reported a proton signal at 5.39 ppm, attached to a carbon at 75.5 ppm with only one additional correlation in the COSY spectrum with a proton at ca. 4.1 ppm, assigned with a hydroxy-methyl carbon at 64.9 ppm in the HSQC spectrum (Figure S7a). The pattern of this unit, labelled **a**, was found to be consistent with that of a Gro unit, phosphorylated at both ends and acylated with an Ala unit at O-2, as described in the WTA polymers containing GroP motifs (43).

Finally, the HSQC spectrum contained three densities at ^1^H/^13^C 4.04/70.8, and 4.12/76.7, labelled as **c_2_**, and **b_2_**, respectively, all identified with the aid of the values reported in literature (Table S3). In detail, **c** was a glycerol unit not further substituted (43), while **b** had the glucose units (**A** and **A’**) linked to O-2 (59). Of note, the HSQC spectrum contained other densities not related to the WTA polymer and presumably belonging to other compounds co-purified with it. In some cases, it was possible to recognize some amino acids, but it was never possible to establish the nature of the compound(s) due to the low intensities of the signals or to the lack of the proper correlations in the full set of NMR spectra acquired. The integration of the **A_5,1_** and **A’_5,1_** densities in the TOCSY spectrum (Figure S7b) revealed that about 15% of this monosaccharide was derivatized with an alanine at O-6.

**REFERENCES**

57. Kontou EE, Walter A, Alka O, Pfeuffer J, Sachsenberg T, Mohite OS, Nuhamunada M, Kohlbacher O, Weber T. 2023. UmetaFlow: an untargeted metabolomics workflow for high-throughput data processing and analysis. J Cheminform 15:52. https://doi.org/10.1186/s13321-023-00724-w

58. Speciale I, Notaro A, Garcia-Vello P, Di Lorenzo F, Armiento S, Molinaro A, Marchetti R, Silipo A, De Castro C. 2022. Liquid-state NMR spectroscopy for complex carbohydrate structural analysis: aA hitchhiker's guide. Carbohydr Polym 277:118885. https://doi.org/10.1016/j.carbpol.2021.118885

59. Garcia-Vello P, Sharma G, Speciale I, Molinaro A, Im S-H, De Castro C. 2020. Structural features and immunological perception of the cell surface glycans of Lactobacillus plantarum: a novel rhamnose-rich polysaccharide and teichoic acids. Carbohydr Polym 233:115857. https://doi.org/10.1016/j.carbpol.2020.115857
